# Metasurface-enabled polarization-independent LCoS spatial light modulator for 4K resolution and beyond

**DOI:** 10.1038/s41377-023-01202-6

**Published:** 2023-06-19

**Authors:** Zhaoxiang Zhu, Yuanhui Wen, Jiaqi Li, Yujie Chen, Zenghui Peng, Jianxiong Li, Lei Zhu, Yunfei Wu, Lidan Zhou, Lin Liu, Liangjia Zong, Siyuan Yu

**Affiliations:** 1 State Key Laboratory of Optoelectronic Materials and Technologies, School of Electronics and Information Technology, Sun Yat-sen University, Guangzhou, 510275 China; 2 Huawei Technologies Co., Ltd., Bantian, Longgang District, Shenzhen, 518129 China; 3 State Key Laboratory of Applied Optics, Changchun Institute of Optics, Fine Mechanics and Physics, Chinese Academy of Sciences, Changchun, 130033 China

**Keywords:** Photonic devices, Integrated optics

## Abstract

With the distinct advantages of high resolution, small pixel size, and multi-level pure phase modulation, liquid crystal on silicon (LCoS) devices afford precise and reconfigurable spatial light modulation that enables versatile applications ranging from micro-displays to optical communications. However, LCoS devices suffer from a long-standing problem of polarization-dependent response in that they only perform phase modulation on one linear polarization of light, and polarization-independent phase modulation—essential for most applications—have had to use complicated polarization-diversity optics. We propose and demonstrate, for the first time, an LCoS device that directly achieves high-performance polarization-independent phase modulation at telecommunication wavelengths with 4K resolution and beyond by embedding a polarization-rotating metasurface between the LCoS backplane and the liquid crystal phase-modulating layer. We verify the device with a number of typical polarization-independent application functions including beam steering, holographical display, and in a key optical switching element - wavelength selective switch (WSS), demonstrating the significant benefits in terms of both configuration simplification and performance improvement.

## Abstract (web summary)

We propose and demonstrate a metasurface-embedded LCoS device that achieves polarization-independent phase modulation at telecommunication wavelengths with 4K resolution and beyond.

## Graphical abstract

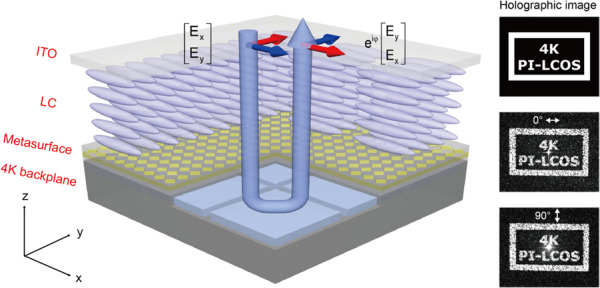

## Introduction

Liquid crystal on silicon (LCoS) spatial light modulator (SLM) is an important optical device that allows on-demand optical wavefront shaping and thus enables versatile functionalities and widespread applications^1,2^, such as microdisplay^3,4^, holography^5^, structured light field^6–8^, optical tweezer^9,10^, optical switching^11–13^, adaptive optics^14^, laser processing^15^, maskless lithography^16^, and additive manufacturing^17^. LCoS devices exploit the birefringence of liquid crystal (LC) materials (versatile LC types including the nematic phase, smectic phase, ferroelectric phase, etc.^1,18,19^) to achieve phase, polarization or amplitude modulation of light driven by the underlying silicon electronics. Phase-only LCoS is especially widely used for wavefront shaping as maximum light conversion efficiency can be achieved. In comparison with other wavefront-shaping SLMs such as deformable mirrors, micro-electro-mechanical systems (MEMS), digital mirror devices (DMD) and liquid crystal displays (LCD), LCoS devices benefit from the combination of efficient light-modulating LC material and mature complementary metal oxide semiconductor (CMOS) silicon backplane, leading to distinct advantages in high spatial resolution (beyond native 4 K), fill factor (>90%), phase modulation resolution (>256-level), reliability and flexibility.

However, a well-known drawback of LCoS devices since their invention is that they only provide phase modulation for one linear polarization that is parallel to the LC director, with almost no effect on the other orthogonal linear polarization. Therefore, existing LCoS devices generally need to be accompanied by polarization-diversity optics in order to modulate the phase of both polarizations simultaneously as often required by most practical applications, leading to high complexity with increased size and cost, as well as performance degradation in terms of polarization-dependent loss (PDL) and polarization mode dispersion (PMD).

In order to solve this long-standing problem, a seemingly straightforward way is to develop new LC materials [such as blue-phase LC^20,21^] with polarization-independent response. However, such LC materials often suffer from high temperature sensitivity and high driving voltage that is incompatible with standard CMOS backplane. Alternatively, two types of schemes have been proposed to modify the conventional polarization-dependent LC. The first scheme integrates two layers of conventional polarization-dependent LC materials with their LC directors orthogonally oriented, so that each LC layer works for one of the two orthogonal linear polarizations^22,23^. This scheme requires a complex LC packaging technique and is especially challenging in precise control of the flatness and thickness of the two LC layers so that they offer exactly the same modulation response to both polarizations. The second one employs a single phase-modulating LC layer and a thin-film quarter wave plate (QWP), which is to be integrated into the CMOS backplane^24^. However, the QWP often has limited working optical bandwidth and its relatively large thickness leads to higher driving voltages and severe fringing field effects^25^, which cannot meet the requirement of a practical polarization-independent (PI) LCoS-SLM device.

**Fig. 1:**
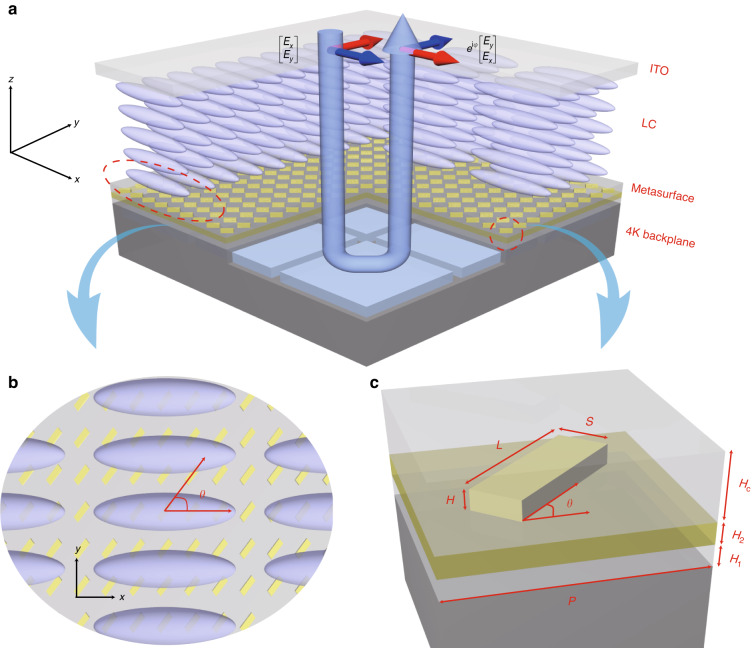
Principle and design of the LCoS device with polarization-independent phase modulation. **a** Schematic of the metasurface-embedded 4 K LCoS device. **b** Top view of the device to indicate the necessary relative orientation angle *θ* = 45° between nanoantenna arrays and LC molecules. **c** The unit cell of the designed Au-insulator-Al (MIM) structure consists of the original LCoS backplane aluminum mirror, passivation layers (H_1_ = 75 nm SiO_2_, H_2_ = 75 nm SiN_x_), a gold nanoantenna and a SiO_2_ cladding layer. The geometric parameters of the nanoantenna and cladding layer are optimized as follows: *L* = 390 nm, *S* = 130 nm, *H* = 70 nm for the nanoantennae, and *H*_C_ = 320 nm for the cladding layer. The unit cells form a square lattice with a period of *P* = 600 nm

We demonstrate, for the first time to our best knowledge, a PI-LCoS-SLM device with 4K resolution. By embedding a well-designed metasurface^26–32^ layer between the original 4K LCoS backplane and LC layer (Fig. 1a), polarization-independent phase modulation is achieved across the targeted C + L telecommunication band. Based on this device, typical application functions including polarization-independent beam steering and holographical imaging are demonstrated with low PDL < 0.3 dB as expected, while other concerned performance (e.g. >40% total efficiency for up to 4^o^ beam-steering angle) being comparable to current commercial polarization-dependent LCoS devices.

## Results

### Concept and design

Polarization-independent phase modulation is realized by inserting an ultrathin metasurface between the LC layer and the backplane (Fig. 1a). The LC layer is responsible for the dynamic phase modulation of the incident light while the metasurface combined with the original backplane forms a metal-insulator-metal (MIM) structure. Each unit cell in this structure acts as a broadband (C + L band), nano-sized reflective half-wave plate (HWP) that performs polarization conversion between the two orthogonal linear polarizations with very high spatial resolution. The principal axes of the LC and the unit cell form an angle of 45° (Fig. 1b), so that the transmission matrix (*T*) of the whole device can be expressed as

$$\begin{array}{c}T=\left(\begin{array}{cc}\exp \left(i\varphi \right) & 0\\ 0 & 1\end{array}\right)\left(\begin{array}{cc}\cos 45^\circ & -\sin 45^\circ \\ \sin 45^\circ & \cos 45^\circ \end{array}\right)\left(\begin{array}{cc}1 & 0\\ 0 & -1\end{array}\right)\left(\begin{array}{cc}\cos 45^\circ & \sin 45^\circ \\ -\sin 45^\circ & \cos 45^\circ \end{array}\right)\left(\begin{array}{cc}\exp \left(i\varphi \right) & 0\\ 0 & 1\end{array}\right)=\exp \left(i\varphi \right)\left(\begin{array}{cc}0 & 1\\ 1 & 0\end{array}\right)\end{array}$$ where $\varphi =2\pi \left({n}_{e}-{n}_{o}\right)d/\lambda$, with $d$ and ${n}_{e}$/${n}_{o}$ being the thickness and extraordinary/ordinary refractive indices of the LC, respectively. The resultant transmission matrix is a simple product of a dynamic phase modulation term and a Pauli matrix, which indicates the desired polarization-independent phase modulation as well as polarization exchange enabled by the metasurface-embedded design. The principle of this polarization-independent operation can be interpreted in a simpler way that the linear-polarized input light after passing through the LC layer is converted to the orthogonal polarization and reflected by the metasurface to again pass through the LC layer backwards, so that each one of the two orthogonal linear polarizations can be equally phase modulated once during the double-pass of the same LC layer.

To realize a PI-LCoS device with high performance, the embedded metasurface layer is critical and should be carefully designed to satisfy the following key requirements: (i) high polarization conversion ratio (PCR), (ii) broadband operation, (iii) insensitivity to the refractive index change of the LC, and (iv) ultrathin thickness to suppress the fringing field effect and minimize any driving voltage rise. With these in mind, we employ a gold/silicon nitride–silicon dioxide/aluminum (Au/SiN_x_–SiO_2_/Al) MIM structure comprising the original backplane and an additional metasurface layer. The metasurface layer consists of Au nanoantennae arranged in a square lattice and a SiO_2_ cladding layer that reduces the interaction between the nanoantennae and the LC while being compatible with the current LC packaging technique. The embedded metasurface layer, simulated and optimized using a finite-difference time-domain (FDTD) method to satisfy the above requirements, has a thickness of only 320 nm (Fig. 1c).

### PCR performance and verification

**Fig. 2:**
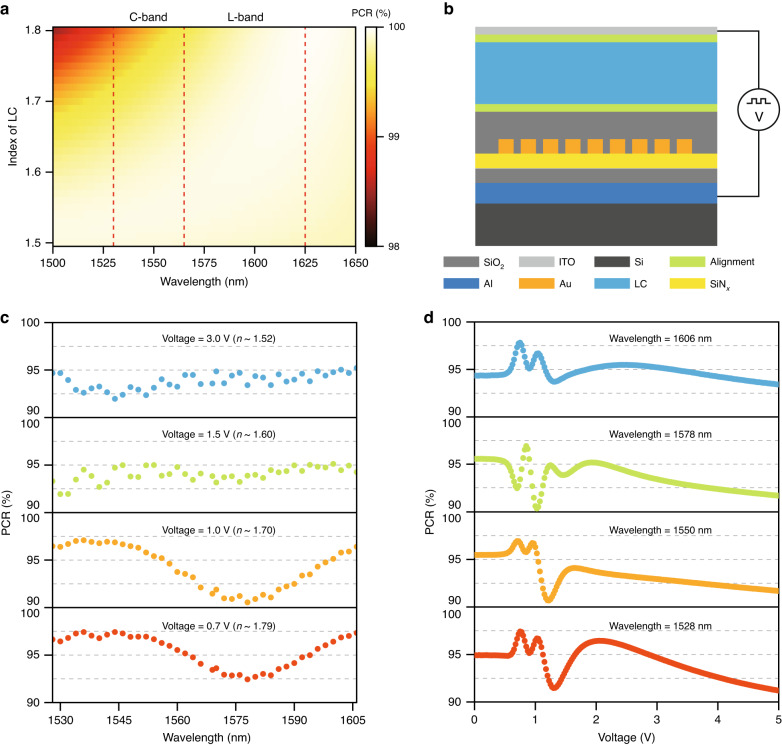
PCR performance and verification based on single-pixel PI-LCoS device. **a** Simulated PCR performance of the device for different wavelengths and refractive index of the LC material. **b** Schematic of the single-pixel PI-LCoS device for experimental verification. Measured PCR performance of the single-pixel device with different driving voltages corresponding to different refractive index of LC (**c**) and working wavelengths among C + L band (**d**). The L-band range is slightly limited by the available laser in the lab

The optimized device is predicted to have a high PCR of >99% across the C + L telecommunication band wavelengths (1530–1625 nm) and over the entire range of LC refractive index modulation (Fig. 2a). To verify this critical performance, we fabricated a single-pixel PI-LCoS device embedded with the designed metasurface (photograph of the device as shown in Supplementary Fig. S6) as shown in Fig. 2b, in which a high birefringent LC material (*n*_e_ ~ 1.81, *n*_o_ ~ 1.5@1550 nm) composed of fluorophenyl and isothiocyanate (NCS) groups with good thermal and photo stability was employed^33–35^. The thickness of the LC layer is around 7.5–8.5 μm to ensure more than 2π phase modulation for both polarizations. The PCR performance of the device is measured at wavelengths across the C + L band and at different applied voltages corresponding to almost the whole LC refractive index modulation range (Fig. 2c, d). The measured PCR is higher than 90% for all cases. The slight degradation compared with simulated predictions (Fig. 2a) is mainly attributed to reflection of the cover glass and fabrication imperfections in the metasurface.

### 4K metasurface-embedded PI-LCoS device and characterization

**Fig. 3:**
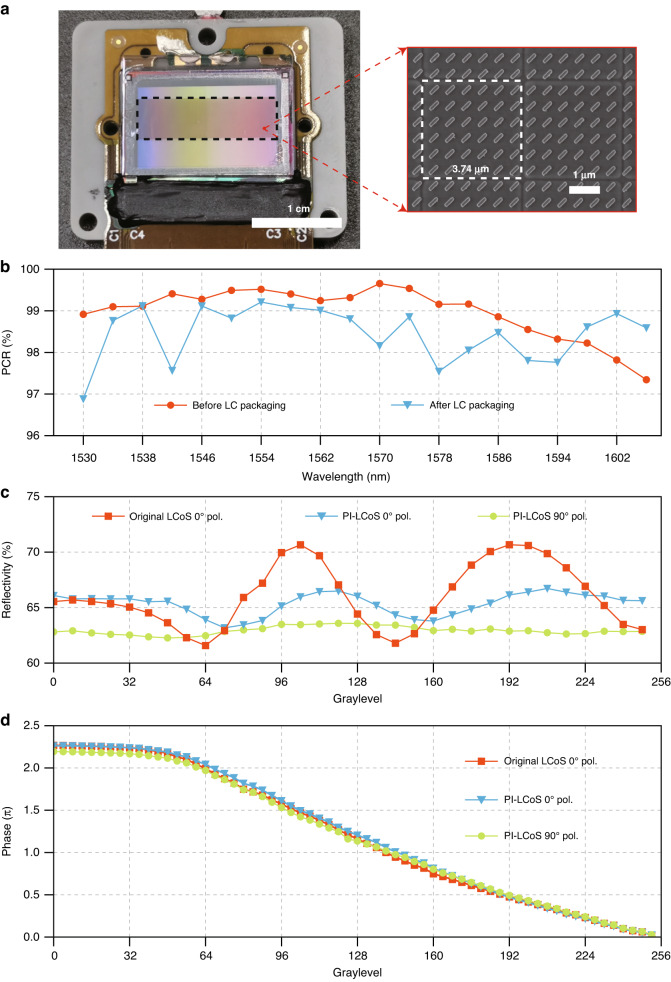
4K metasurface-embedded PI-LCoS device and its critical characteristics. **a** Photograph of the fabricated 4 K LCoS device (4160 × 2464 pixels), in which half of the area (4160 × 1232 pixels with 3.74 μm pixel size) as indicated in the black dashed box has embedded metasurface, so that performance of the PI-LCoS (area with metasurface) and original LCoS (area without metasurface) can be compared. An enlarged scanning electron microscope (SEM) image of the nanoantenna array is presented on the right. **b** Measured PCR performance of the PI-LCoS before and after LC packaging. Measured reflectivity (**c**) and phase response (**d**) of the original LCoS with 0° (LC alignment direction) linear-polarized incident light and PI-LCoS with both 0° and 90° linear-polarized incident light. The amount of phase shift in the original LCoS is halved for better comparison with that of the PI-LCoS

With the verification of the metasurface performance, we further apply the structure to a commercial 4K backplane (JD2704 Microdisplay Wafer, Jasper Display Corp.) to produce a 4K metasurface-embedded LCoS device and characterize its critical performances. The fabricated 4K LCoS device (4160 × 2464 pixels with 3.74 μm pixel size) with the embedded metasurface is shown in Fig. 3a. Half of the backplane area (4160 × 1232 pixels) has been embedded metasurface as indicated by the dashed box (Fig. 3a, inset), so that the performance of the PI-LCoS (the area with metasurface) can be compared with that of the original LCoS (the area without metasurface).

PCR performance is firstly measured before and after LC packaging (Fig. 3b), which again confirms efficient polarization conversion of >96%. Reflectivity measurement of the device is then performed by displaying uniform 0–255 phase levels in succession, corresponding to the applied voltage ranging from 0.7 V to 3.5 V. The measured average reflectivity of the original LCoS and the PI-LCoS are 66.0% and 64.1% (0^o^ polarization: 62.9%, 90^o^ polarization: 65.3%), respectively (Fig. 3c). The difference of only ~2% indicates almost negligible loss caused by the embedded metasurface. The electro-optic phase responses of the original LCoS and the PI-LCoS are both characterized based on a standard binary grating method, which employs a binary grating hologram with two graylevels of 0 (fixed) and GL (varying from 0–255) displayed on the PI-LCoS device and measured the optical power of the first order in the far-field diffraction. Based on this, the phase modulation amount of the device can be derived from the measured optical power, and thus the relationship between the displayed graylevel and the phase response is obtained as shown in Fig. 3d. The result shows that PI-LCoS has almost the same phase responses for both 0° and 90° linear-polarized light, which verifies the targeted polarization-independent phase modulation. As expected, the amount of phase modulation for the PI-LCoS almost coincides with half of that for the original LCoS, which indicates the embedded ultrathin metasurface structure causes negligible deterioration in the driving voltage and fringing field effect.

### Beam steering and holographic imaging

To verify the high-performance polarization-independent phase modulation by the fabricated PI-LCoS device, two widely used functionalities—beam steering and holographic imaging—are demonstrated at the wavelength of 1550 nm.

**Fig. 4:**
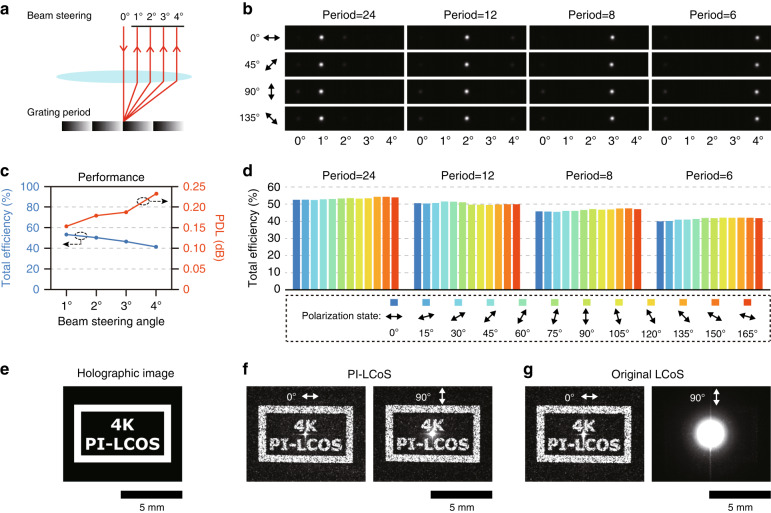
Demonstration of two basic functionalities using the fabricated PI-LCoS device. **a**–**d** Schematic of beam steering (**a**) and the measured optical intensity distributions of different linearly polarized light after beam steering with diffraction angles from 1° to 4° (**b**). All images have the same intensity reference and position reference. Quantitative analysis of the beam-steering performance with total efficiency and polarization dependence (**c**) for different linearly polarized light with polarization angles at every 15° between 0° to 180° (0° corresponds to the direction of LC directors) (**d**). Holographic imaging of ‘4 K PI-LCOS’ logo (**e**) by the PI-LCoS (**f**) and the original LCoS (**g**) under two orthogonal linearly polarized input light (0° and 90° polarization). The scale bar for these holographic images is 5 mm in size

For the beam-steering operation, different diffraction angles up to 4° are realized by imposing blaze grating images with corresponding grating periods. The steering angles are mapped to different horizontal positions in the focal plane of a lens (Fig. 4a). Linearly polarized light beams with polarization angles at every 15° between 0°-180° are incident onto the device in succession. For each grating period, different polarizations are steered to the same position with similar optical intensity profiles (Fig. 4b). Here the total efficiency including both background reflectance and diffraction loss is employed to better characterize the whole performance of the device between the input and the output light as well as the concerned PDL performance. Different linearly polarized light beams have almost the same total efficiency at the same diffraction angle, with the PDL being below 0.3 dB (~6.7%) up to the diffraction angle of 4° (Fig. 4c, d). Quantitatively, the total efficiency averaged on different polarization states is 53.2%, 50.3%, 46.5% and 41.4% for 1°, 2°, 3° and 4° beam steering, respectively (Fig. 4c), which is comparable with the performance of commercial LCoS devices (for instance, GAEA-2-TELCO-033, Holoeye) based on the same 4 K backplane.

In addition to beam steering, the polarization-independent holographic imaging functionality is also verified and presented in Fig. 4e, in which a ‘4K PI-LCOS’ logo is imaged through computer-generated holography (CGH) that is designed based on the standard Gerchberg-Saxton algorithm^5^. For the PI-LCoS (Fig. 4f), holographic imaging can be generated by the CGH for both orthogonal linear polarizations, while the original LCoS (Fig. 4g) can only work at one of the two polarizations (0° polarization) with almost no effect on the orthogonal polarization (90° polarization).

### A practical application in optical switching

**Fig. 5:**
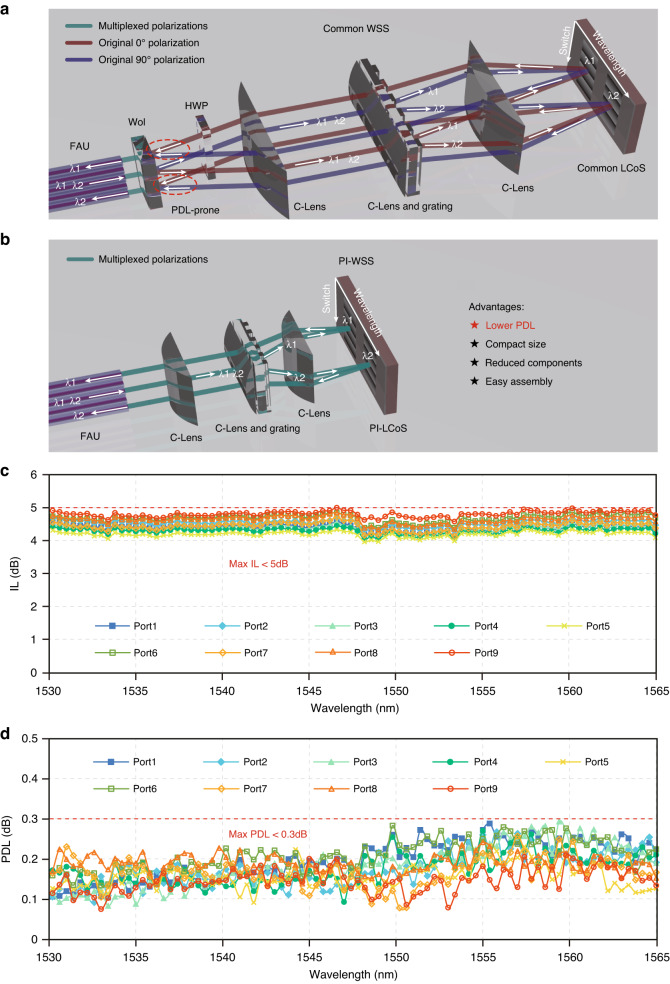
Demonstration of a 1 × 9 PI-WSS module based on PI-LCoS. Illustrations of a conventional 1 × 9 WSS based on an ordinary LCoS device (**a**) and an 1 × 9 PI-WSS based on the PI-LCoS device (**b**). FAU fiber array unit, Wol Wollaston prism, HWP half-wave plate, C-lens cylindrical lens. Performance of the assembled PI-WSS including IL (**c**) and PDL (**d**) for all the nine output ports in the C band

To further demonstrate the benefits of the PI-LCoS device in practical applications, we apply it as the core beam-steering engine in a wavelength selective switch (WSS) module. The WSS is a key optical component widely deployed in modern wavelength division multiplexed (WDM) optical fiber communication networks for large-capacity optical signal routing. LCoS has replaced MEMS as the central beam-steering engine in WSS^36^ to enable flex-grid wavelength routing in optical network. However, compared with MEMS switches, LCoS has been polarization-dependent and thus less compatible with optical fiber communication systems where the signal polarization upon arriving at the WSS is uncertain and can even be polarization-multiplexed with both polarizations carrying data. Therefore, current LCoS-based WSS modules always require elaborate polarization-diversity optics (Fig. 5a), leading to complicated optical designs with large number of bulk optical components, and inferior PDL performance^11^. The PI-LCoS demonstrated in this work significantly simplifies the WSS modules (Fig. 5b) by allowing both orthogonal polarizations to follow a single optical path, eliminating: (i) the off-axis effect hindering more compact size and (ii) PDL arising during polarization separation. Moreover, the PI-LCoS also ‘swaps’ the two orthogonal linear polarizations, which can be exploited to further compensate the PDL introduced by other optical components in the WSS module.

For verification, a compact PI-WSS module is assembled and characterized. The measured insertion loss (IL) is less than 5 dB (fiber–fiber) for all the output ports across the C band (Fig. 5c), while the measured PDL is less than 0.3 dB for all cases (Fig. 5d), which is significantly reduced compared with the ~1 dB PDL of existing LCoS-based WSS. Therefore, the proposed PI-WSS realizes flex-grid wavelength selective switching and meanwhile maintaining low PDL that is comparable with MEMS-based WSS.

## Discussion

We propose and demonstrate a metasurface-embedded LCoS device that achieves polarization-independent spatial phase modulation with 4K resolution across C + L telecommunication band. The integration of the metasurface allows a significant reduction of the thickness required for high-efficient polarization conversion and enables optically broadband operation free of noticeable deterioration on fringing field effect, which is critical for the realization of PI-LCoS with high performance comparable to commercial LCoS-SLM products. The demonstrated PI-LCoS device is further applied to practical application scenarios including beam steering, holographic display, as well as the crucial beam-steering engine in a wavelength selective switch for telecommunications, manifesting significant benefits in configuration simplification and PDL performance improvement. Our scheme can be readily extended to different operation wavelengths (e.g., for visible display applications) and LCoS backplanes with various resolutions and pixel sizes, while the small size of the unit-cell supports future upgrade toward 8 K resolution. Such a metasurface-embedded PI-LCoS is expected to significantly impact on a variety of applications bringing ample values in higher performance, lower complexity, and lower cost.

## Materials and methods

### Design

The metasurface-embedded PI-LCoS device is designed and optimized by lumerical finite-difference time-domain (FDTD) software. According to the 4K backplane, the simulated substrate is composed of silicon (Si), 260 nm aluminum (Al), 75 nm silica (SiO_2_) and 75 nm silicon nitride (SiNx). To realize high-performance PI-LCoS device, the parameters of Au nanoantenna (L, S and H, Fig. 1c) and cladding layer (*H*_C_) need to be optimized in order to achieve the polarization conversion ratio (PCR) over 99% with different refractive index of LC (*n* = 1.5–1.8). The PCR is defined by ${\rm{PCR}}={P}_{\rm{c}}/\left({P}_{\rm{c}}+{P}_{{\rm{uc}}}\right)$ for the case of incident 0° linear polarization light, where ${P}_{\rm{c}}$ and ${P}_{{\rm{uc}}}$ are the optical power of converted (90°) and unconverted (0°) linear polarization light, respectively. The optimized metasurface layer has an ultrathin thickness of only 320 nm, with parameters of L = 390 nm, S = 130 nm, H = 70 nm and H_C_ = 320 nm.

### Fabrication

The single-pixel PI-LCoS with embedded metasurface (Fig. S7) is fabricated as follows. On the silicon (Si) wafer, a 260-nm aluminum (Al) film and a 75-nm SiO_2_ film are deposited by the Leybold APS1104 ion-assisted deposition system, and then a 75-nm SiN_*x*_ film is deposited by the Oxford PlasmaPro System100 ICP180-CVD inductively coupled plasma chemical vapor deposition system (ICPCVD) (Fig. S7a). These three films are deposited to simulate a 4K LCoS backplane. After cleaning (sequentially sonicate in acetone and isopropanol solutions for 10 min) and drying (at 130 °C for 5 min), lift-off resist (LOR, 3A, MicroChem Inc.) and positive tone electron-beam resist polymethyl methacrylate (PMMA, A7, Kayaku Advanced Materials Inc.) are spin-coated sequentially as masks (Fig. S7b). The use of bilayer resist is to avoid metal residues during subsequent metal deposition. Electron-beam lithography (EBL) is then performed to define the masks for the nanoantenna arrays using Raith EBPG5000 + EBL system, followed by sequential immersion in 4-Methyl-2-pentanone (MIBK), isopropyl alcohol (IPA), LOR developer (positive photoresist developers), and deionized water to develop and fix the nanoantenna arrays pattern (Fig. S7c). Then, 3 nm titanium (Ti), 70 nm aurum (Au), and 3 nm Ti are successively deposited by electron-beam evaporator (DE400), and two layers of Ti are both used to increase the adhesion of Au (Fig. S7d). After soaking the sample in acetone to remove the remaining resist (Fig. S7e), a 1.5-μm SiO_2_ film is deposited again with the ICPCVD system as a cladding layer (Fig. S7f). The thickness of cladding layer exceeds our designed value in order to flatten the uneven surface caused by nanoantenna arrays. To obtain the designed thickness precisely, multiple etching steps of the device is performed by a reactive ion etching system (RIE, PlasmaPro System 100RIE). The optical setup of Fig. S10 is used to measure the PCR performance of the device after each etching step, and it is terminated when the PCR value reached the required characteristics as design (Fig. S7g). Then, an alignment layer is coated on the flattened cladding layer and rubbed for alignment (Fig. S7h), while non-contact photoalignment^37^ might also be employed in future for better alignment. Finally, the device is packaged with LC (Fig. S7i) and wire bonded. The detailed process of LC packaging is shown in Fig. S8. The 4 K PI-LCoS based on commercial 4 K backplane is also produced in a similar process, except that exposed electrode pads of the backplane need to be carefully protected during the whole fabrication process.

### Optical characterization

An optical measurement setup is built as shown in Fig. S10 to characterize the performance of the device. In the experiment, we use a 1 × 2 fiber coupler with a power ratio of 50:50 to combine the C- and L-band tunable laser modules and only one laser module is turned on during the test. After the beam is collimated by the collimator, it continuously passes through two linear polarizers (P1 and P2, Thorlabs, Inc.) to ensure that the beam emitted from the tunable laser has a high degree of the linear polarization. Here, connecting a polarization controller (PC) enables flexibly control of the beam’s optical power. The HWP is used to rotate the linear polarization angle, and P3 is also used to ensure a high degree of polarization for the linearly polarized light. Lens1 and Lens2 form a 4f system to achieve beam size scaling. The sample is placed on one arm of the beam splitter (BS) and the QWP and mirror are placed on the other arm (as indicated by the red dashed box), which forms a Michelson interferometer. Assisted with a camera, the optical setup can be used to measure the phase modulation amount of the sample deduced from the movement of the interference fringe. The insertion of QWP is to align the linear polarization states of the two beams. When the optical elements in the red dashed box are removed, the PCR of the sample can be calculated based on the optical power of 0° and 90° linear polarizations measured by a photodetector (PD, S122C, Thorlabs Inc.) for incident 0^o^ linear polarization light.

## Supplementary information

Supplementary Information for Metasurface-enabled Polarization Independent LCoS Spatial Light Modulator for 4K Resolution and Beyond

## References

[1] Zhang ZC, You Z, Chu DP (2014). Fundamentals of phase-only liquid crystal on silicon (LCOS) devices. Light Sci. Appl..

[2] Lazarev G (2019). Beyond the display: phase-only liquid crystal on Silicon devices and their applications in photonics. Opt. Express.

[3] Vettese D (2010). Microdisplays: liquid crystal on silicon. Nat. Photonics.

[4] Yin K (2022). Advanced liquid crystal devices for augmented reality and virtual reality displays: principles and applications. Light Sci. Appl..

[5] Blanche PA (2021). Holography, and the future of 3D display. Light Adv. Manuf..

[6] Cai XL (2012). Integrated compact optical vortex beam emitters. Science.

[7] Wen YH (2018). Spiral transformation for high-resolution and efficient sorting of optical vortex modes. Phys. Rev. Lett..

[8] Forbes A, De Oliveira M, Dennis MR (2021). Structured light. Nat. Photonics.

[9] Dholakia K, Čižmár T (2011). Shaping the future of manipulation. Nat. Photonics.

[10] Gieseler J (2021). Optical tweezers — from calibration to applications: a tutorial. Adv. Opt. Photonics.

[11] Ma YR (2021). Recent progress of wavelength selective switch. J. Lightw. Technol..

[12] Wang M (2017). LCoS SLM study and its application in wavelength selective switch. Photonics.

[13] Marom DM (2017). Survey of photonic switching architectures and technologies in support of spatially and spectrally flexible optical networking. J. Opt. Commun. Netw..

[14] Hampson KM (2021). Adaptive optics for high-resolution imaging. Nat. Rev. Methods Primers.

[15] Salter PS, Booth MJ (2019). Adaptive optics in laser processing. Light Sci. Appl..

[16] Jenness NJ (2010). A versatile diffractive maskless lithography for single-shot and serial microfabrication. Optics Express.

[17] Shusteff M (2017). One-step volumetric additive manufacturing of complex polymer structures. Sci. Adv..

[18] Srivastava AK, Chigrinov VG, Kwok HS (2015). Ferroelectric liquid crystals: excellent tool for modern displays and photonics. J. Soc. Info. Disp..

[19] Chen X (2020). First-principles experimental demonstration of ferroelectricity in a thermotropic nematic liquid crystal: Polar domains and striking electro-optics. Proc. Natl Acad. Sci. USA.

[20] Meiboom S (1981). Theory of the blue phase of cholesteric liquid crystals. Phys. Rev. Lett..

[21] Hyman RM (2014). Polarization-independent phase modulation using a blue-phase liquid crystal over silicon device. Appl. Opt..

[22] Lin YH (2005). Polarization-independent liquid crystal phase modulator using a thin polymer-separated double-layered structure. Opt. Express.

[23] He ZQ (2017). Polarization-independent phase modulators enabled by two-photon polymerization. Opt. Express.

[24] Moore JR (2008). The silicon backplane design for an LCOS polarization-insensitive phase hologram SLM. IEEE Photonics Technol. Lett..

[25] Apter B, Efron U, Bahat-Treidel E (2004). On the fringing-field effect in liquid-crystal beam-steering devices. Appl. Opt..

[26] Dorrah AH, Capasso F (2022). Tunable structured light with flat optics. Science.

[27] Li SQ (2019). Phase-only transmissive spatial light modulator based on tunable dielectric metasurface. Science.

[28] Dorrah AH (2021). Metasurface optics for on-demand polarization transformations along the optical path. Nat. Photonics.

[29] Xie YY (2020). Metasurface-integrated vertical cavity surface-emitting lasers for programmable directional lasing emissions. Nat. Nanotechnol..

[30] Joo WJ (2020). Metasurface-driven OLED displays beyond 10, 000 pixels per inch. Science.

[31] Zhao Y, Alù A (2013). Tailoring the dispersion of plasmonic nanorods to realize broadband optical meta-waveplates. Nano Lett..

[32] Gu T (2023). Reconfigurable metasurfaces towards commercial success. Nat. Photonics.

[33] Peng ZH (2016). Electrooptical properties of new type fluorinated phenyl-tolane isothiocyanate liquid crystal compounds. Liq. Cryst..

[34] Gauza S (2005). High birefringence and high resistivity isothiocyanate-based nematic liquid crystal mixtures. Liq. Cryst..

[35] Khoo, I. *Liquid Crystals* (John Wiley & Sons, Hoboken, 2022).

[36] Lord, A. et al. Evolution from wavelength-switched to flex-grid optical networks. In *Elastic Optical Networks* (eds. López, V. & Velasco, L.) 7–30 (Springer,Cham, 2016).

[37] Chigrinov, V. G., Kozenkov, V. M. & Kwok, H. S. *Photoalignment of Liquid Crystalline Materials: Physics and Applications* (John Wiley & Sons, Hoboken, 2008).

