# Supplementary material for: Metasurface-enabled polarization-independent LCoS spatial light modulator for 4K resolution and beyond
Source: Light Sci Appl. 2023 Jun 19;12:151. doi: 10.1038/s41377-023-01202-6 (PMC10277279; doi:10.1038/s41377-023-01202-6)
Supplement: Supplementary file 1 — Supplementary Information for Metasurface-enabled Polarization Independent LCoS Spatial Light Modulator for 4K Resolution and Beyond [file 41377_2023_1202_MOESM1_ESM.docx]

Supplementary Information for

Metasurface-enabled Polarization Independent LCoS Spatial Light Modulator for 4K Resolution and Beyond

Zhaoxiang Zhu^1†^, Yuanhui Wen^2†^, Jiaqi Li^1†^, Yujie Chen^1‡^, Zenghui Peng^3^, Jianxiong Li^2^, Lei Zhu^2^, Yunfei Wu^2^, Lidan Zhou^1^, Lin Liu^1^, Liangjia Zong^2‡^, Siyuan Yu^1^

^1^State Key Laboratory of Optoelectronic Materials and Technologies, School of Electronics and Information Technology, Sun Yat-sen University, Guangzhou 510275, China.

^2^Huawei Technologies Co., Ltd., Bantian, Longgang District, Shenzhen 518129, China.

^3^State key laboratory of applied optics, Changchun Institute of Optics, Fine Mechanics and Physics, Chinese Academy of Sciences, Changchun 130033, China.

^‡^**Corresponding author:** chenyj69@mail.sysu.edu.cn (Y. C.);

zongliangjia@huawei.com (L.-J. Z.)

†These authors contributed equally to this work.

**Design and parameter optimization of the** **metasurface-embedded polarization-independent LCoS device.** We employ finite-difference time-domain (FDTD) method to design the metasurface-embedded polarization-independent (PI) LCoS device and optimize its performance. Critical parameters of the device as indicated in Fig. S1a, including the length and width of the nanoantenna arrays as well as the thickness of the cladding layer, are optimized as shown in Fig. S1 and Fig. S2. Figure S1, b and c show that the optimized length and width of the nanoantenna unit are 390 nm and 130 nm, respectively, while the thickness of the cladding layer is 320 nm. It is also noted that the device maintains broadband high polarization conversion ratio (PCR) performance when the refractive index of the LC layer varies from 1.5 to 1.8. Figure S2 shows that the optimized thickness of the nanoantenna array and the period of unit cell, and Fig. S3 shows that the optimized thickness of the cladding layer. The PCR performance of the device is not sensitive to the thickness of the SiO_2_ cladding layer when the refractive index of the LC layer is 1.5. While the refractive index is changed to 1.0 and 1.8, the PCR of the device related to the thickness of the cladding layer has the same variation trend, which can be employed to determine the optimized thickness cladding layer in experiment even without LC packaging yet.


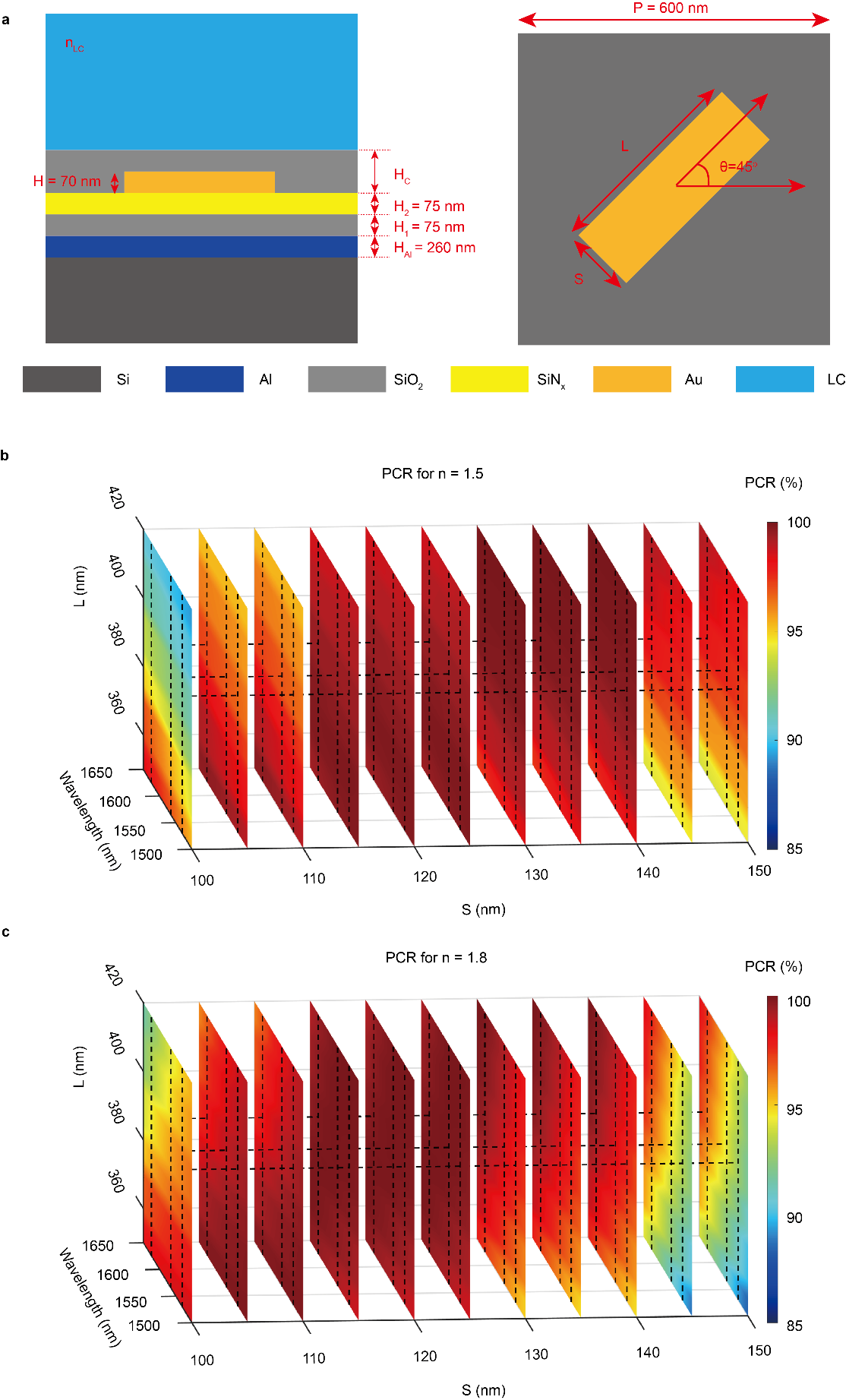


**Fig. S1. a** Schematic of the unit cell of metasurface-embedded LCoS device (cross-section on the left and top view on the right). Numerical PCR performance of the metasurface-embedded LCoS device for LC refractive indices of **b** n = 1.5 and **c** n = 1.8 with the cladding layer thickness of 320 nm. L and S refer to the length and width of the nanoantenna unit, while the thickness and period of the nanoantenna unit are H = 70 nm and P = 600 nm, respectively. The vertical black dotted lines indicate the boundaries of C+L band while the horizontal black dotted lines are the boundaries of C+L band for the case of optimized L = 390 nm.


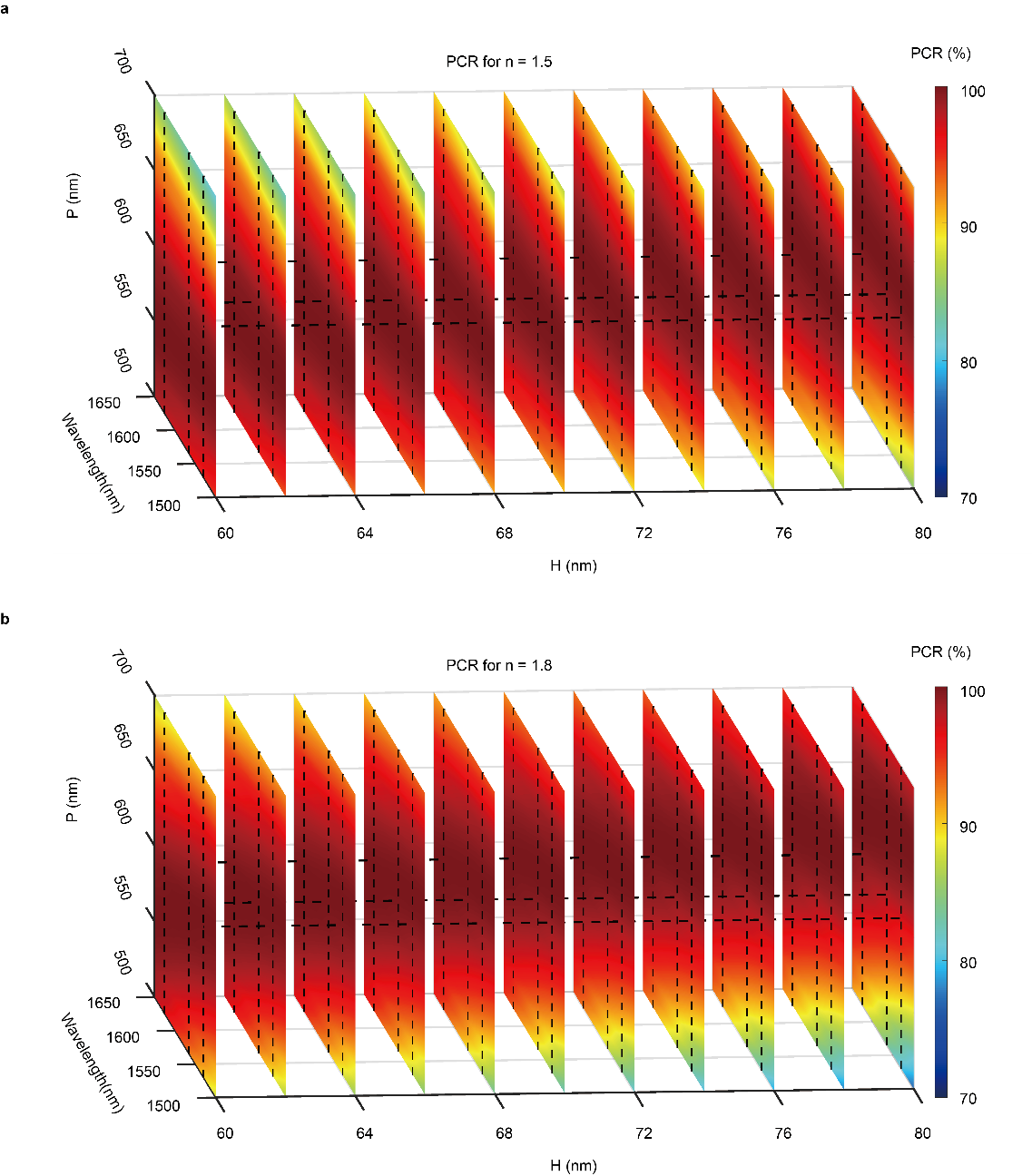


**Fig. S2.** Numerical PCR performance of the metasurface-embedded LCoS device for LC refractive indices of **a** n = 1.5 and **b** n = 1.8 with the cladding layer thickness of 320 nm. P and H refer to the period of the unit cell and the thickness of the nanoantenna array, while the length and width of the nanoantenna unit are L = 390 nm and S = 130 nm, respectively. The vertical black dotted lines indicate the boundaries of C+L band while the horizontal black dotted lines are the boundaries of C+L band for the case of optimized P = 600 nm.


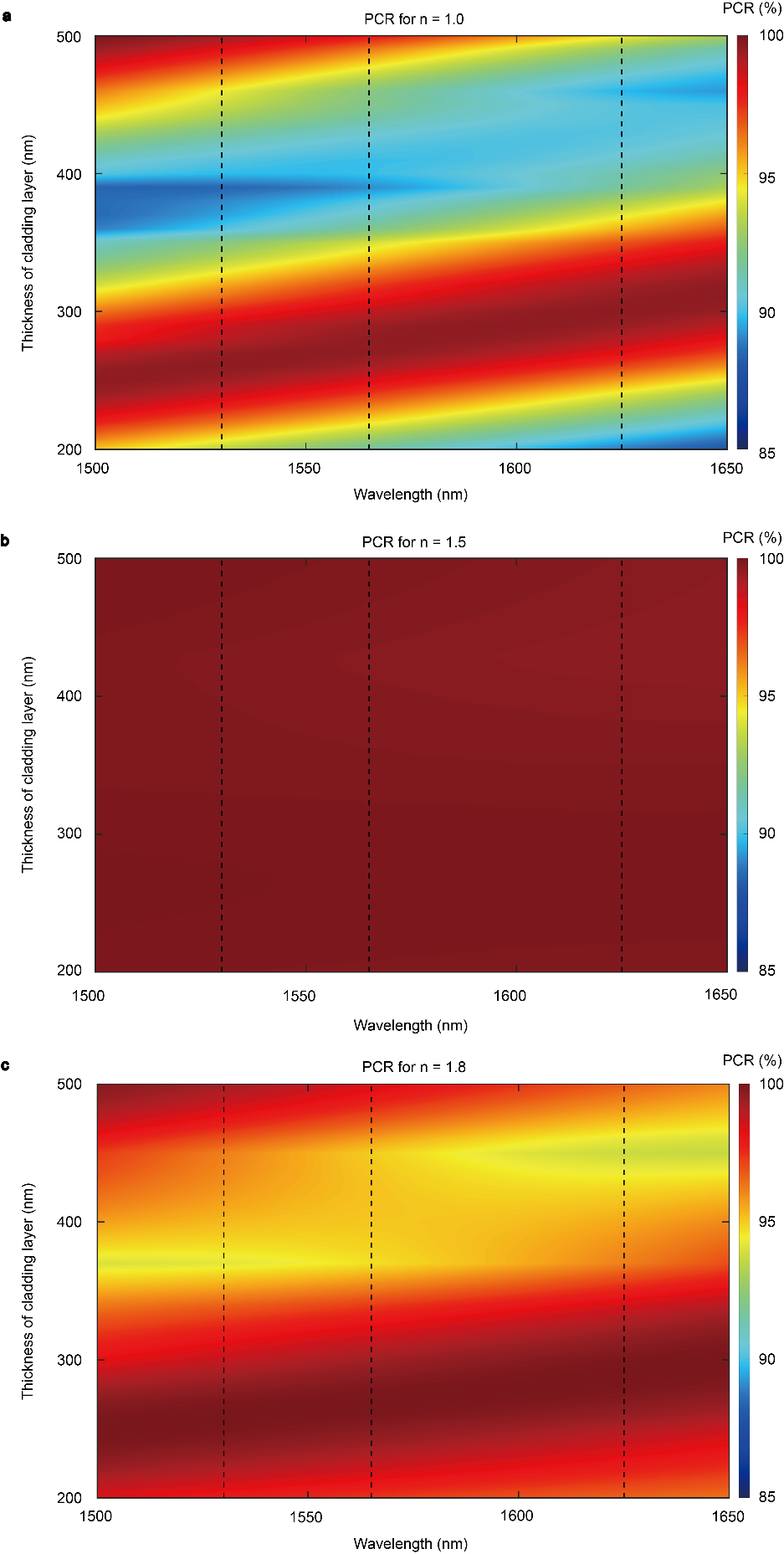


**Fig. S3.** Numerical PCR perfomance of the metasurface-embedded LCoS device for LC refractive indices of **a** n =1.0, **b** n = 1.5 and **c** n = 1.8. Nanoantenna array parameters are L = 390 nm, S = 130 nm, H = 70nm and P = 600 nm. The vertical black dotted lines indicate the boundaries of C+L band.

**The evaluation of the error of the alignment of the unit-cell to the LC axis.** We have added a set of cross markers during the fabrication of the metasurface structure for subsequent alignment in LC packaging, which are in length of $l=1 mm$ and in width of $w=50 \mu m$ as shown in Fig. S4a. The distance between adjacent markers is $d_{M}=11.5 mm$ (see Fig. S4b). These markers and the metasurface structure are fabricated simultaneously in the EBL process. Noted that the long side of the metasurface region is parallel to the transverse direction of the cross marker.


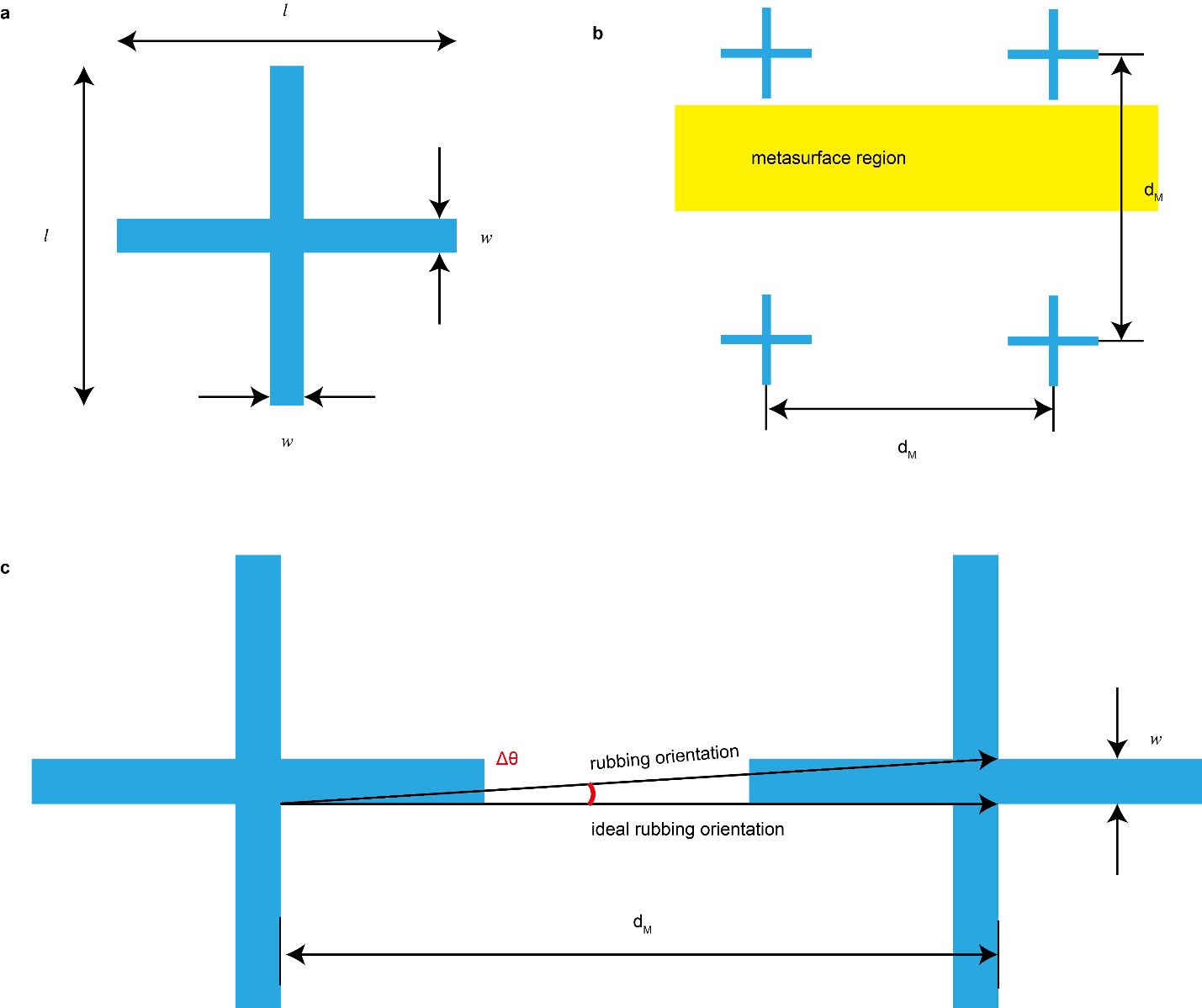


**Fig. S4.** Schematic of the alignment markers. **a** Illustration of single marker, the length of which is $l=1 mm$ while the width is $w=50 \mu m$. **b** Illustration of the markers’ relative location, where metasurface is located in the middle of markers. **c** Illustration of the angle error of LC rubbing orientation in alignment process.

The alignment process of LC packaging is carried out under a microscope, so the rubbing orientation can be accurately performed based on the direction of the marker's line (ideal rubbing orientation), as shown in Fig. S4c. Theoretically, the formulate for the angle error between the liquid crystal orientation and the transverse direction of the metasurface can be written as:

$$\begin{aligned} \Delta\theta=arctan\left( \frac{w}{d_{M}} \right)=\arctan\left( \frac{0.05}{11.5} \right)\approx0.0043 rad \left( \approx0.25^{\circ} \right)\#\left( s = 1 \backslash* Arabic 1 \right) \end{aligned}$$

Therefore, the error of the angle between the unit cell of the metasurface and LC can be controlled within $\pm0.25^{\circ}$ in principle.

Suppose the angle between the axes of the unit cell and LC is$x$, then the transmission matrix can be written as:

$$\begin{aligned} T&=\left( \begin{matrix} exp\left( i\varphi\right) & 0 \\ 0 & 1 \end{matrix} \right)\left( \begin{matrix} cos\left( x \right) & -sin\left( x \right) \\ sin\left( x \right) & cos\left( x \right) \end{matrix} \right)\left( \begin{matrix} 1 & 0 \\ 0 & -1 \end{matrix} \right)\left( \begin{matrix} cos\left( x \right) & sin\left( x \right) \\ -sin\left( x \right) & cos\left( x \right) \end{matrix} \right)\left( \begin{matrix} exp\left( i\varphi\right) & 0 \\ 0 & 1 \end{matrix} \right) \\ &=\left( \begin{matrix} exp\left( i\varphi\right) & 0 \\ 0 & 1 \end{matrix} \right)\left( \begin{matrix} cos\left( x \right) & -sin\left( x \right) \\ sin\left( x \right) & cos\left( x \right) \end{matrix} \right)\left( \begin{matrix} 1 & 0 \\ 0 & -1 \end{matrix} \right)\left( \begin{matrix} cos\left( x \right)exp\left( i\varphi\right) & sin\left( x \right) \\ -sin\left( x \right)exp\left( i\varphi\right) & cos\left( x \right) \end{matrix} \right) \\ &=\left( \begin{matrix} exp\left( i\varphi\right) & 0 \\ 0 & 1 \end{matrix} \right)\left( \begin{matrix} cos\left( x \right) & -sin\left( x \right) \\ sin\left( x \right) & cos\left( x \right) \end{matrix} \right)\left( \begin{matrix} cos\left( x \right)exp\left( i\varphi\right) & sin\left( x \right) \\ sin\left( x \right)exp\left( i\varphi\right) & -cos\left( x \right) \end{matrix} \right) \\ &=\left( \begin{matrix} exp\left( i\varphi\right) & 0 \\ 0 & 1 \end{matrix} \right)\left( \begin{matrix} \left( {cos}^{2}\left( x \right)-{sin}^{2}\left( x \right) \right)exp\left( i\varphi\right) & 2sin\left( x \right)cos\left( x \right) \\ 2sin\left( x \right)cos\left( x \right)exp\left( i\varphi\right) & -{cos}^{2}\left( x \right)+{sin}^{2}\left( x \right) \end{matrix} \right) \\ &=\left( \begin{matrix} exp\left( i\varphi\right) & 0 \\ 0 & 1 \end{matrix} \right)\left( \begin{matrix} cos\left( 2x \right)exp\left( i\varphi\right) & sin\left( 2x \right) \\ sin\left( 2x \right)exp\left( i\varphi\right) & -cos\left( 2x \right) \end{matrix} \right) \\ &=\left( \begin{matrix} cos\left( 2x \right)exp\left( i2\varphi\right) & sin\left( 2x \right)exp\left( i\varphi\right) \\ sin\left( 2x \right)exp\left( i\varphi\right) & -cos\left( 2x \right) \end{matrix} \right)\#\left( s = 2 \backslash* Arabic 2 \right) \end{aligned}$$

When the angle is $x=\frac{\pi}{4}$ in the ideal case, the transmission matrix for the device can be written as:

$\begin{aligned} T\left( x=\frac{\pi}{4} \right)&=\left( \begin{matrix} cos\left( 2x \right)exp\left( i2\varphi\right) & sin\left( 2x \right)exp\left( i\varphi\right) \\ sin\left( 2x \right)exp\left( i\varphi\right) & -cos\left( 2x \right) \end{matrix} \right)& \\ &=\left( \begin{matrix} 0 & exp\left( i\varphi\right) \\ exp\left( i\varphi\right) & 0 \end{matrix} \right) \\ &=exp\left( i\varphi\right)\left( \begin{matrix} 0 & 1 \\ 1 & 0 \end{matrix} \right)\#\#\left( s = 3 \backslash* Arabic 3 \right) \end{aligned}$If the angle $x$ slightly deviates from $\frac{\pi}{4}$, we set $x_{0}=\frac{\pi}{4}$ and the variation of the angle is $\Delta x$, the transmission matrix in Eq. (s2) can be written as:

$$\begin{aligned} T\left( x=x_{0}+\Delta x \right)=\left( \begin{matrix} cos\left( 2\left( x_{0}+\Delta x \right) \right)exp\left( i2\varphi\right) & sin\left( 2\left( x_{0}+\Delta x \right) \right)exp\left( i\varphi\right) \\ sin\left( 2\left( x_{0}+\Delta x \right) \right)exp\left( i\varphi\right) & -cos\left( 2\left( x_{0}+\Delta x \right) \right) \end{matrix} \right)\#\left( s = 4 \backslash* Arabic 4 \right) \end{aligned}$$

According to the Taylor formula, trigonometric functions can be expanded around the angle $x_{0}$ as:

$$\begin{aligned} cos\left( 2\left( x_{0}+\Delta x \right) \right)=cos\left( 2x_{0} \right)-2sin\left( 2x_{0} \right)\Delta x+o\left( \left( \Delta x \right)^{2} \right)\#\left( s5 \right) \end{aligned}$$

$$\begin{aligned} sin\left( 2\left( x_{0}+\Delta x \right) \right)=sin\left( 2x_{0} \right)+2cos\left( 2x_{0} \right)\Delta x+o\left( \left( \Delta x \right)^{2} \right)\#\left( s = 6 \backslash* Arabic 6 \right) \end{aligned}$$

Because the high-order terms can be ignored, the transmission matrix of the device can be derived as below:

$$\begin{aligned} T\left( x=x_{0}+\Delta x \right) \\ &=\left( \begin{matrix} \left( cos\left( 2x_{0} \right)-2sin\left( 2x_{0} \right)\Delta x \right)exp\left( i2\varphi\right) & \left( sin\left( 2x_{0} \right)+2cos\left( 2x_{0} \right)\Delta x \right)exp\left( i\varphi\right) \\ \left( sin\left( 2x_{0} \right)+2cos\left( 2x_{0} \right)\Delta x \right)exp\left( i\varphi\right) & -cos\left( 2x_{0} \right)+2sin\left( 2x_{0} \right)\Delta x \end{matrix} \right)\#\left( s = 7 \backslash* Arabic 7 \right) \end{aligned}$$

According to Eq. (s7) with $x_{0}=\frac{\pi}{4}$, the transmission matrix can be written by

$$\begin{aligned} T\left( x=45^{\circ}+\Delta x \right)=\left( \begin{matrix} -2\Delta xexp\left( i2\varphi\right) & exp\left( i\varphi\right) \\ exp\left( i\varphi\right) & 2\Delta x \end{matrix} \right)\#\left( s8 \right) \end{aligned}$$

Based on Eq. (s8), the output light field can be expressed as Eqs. (s9) and (s10) for input x- and y- polarized light, respectively.

$$\begin{aligned} E_{out}^{x}=TE_{x}=\left( \begin{matrix} -2\Delta xexp\left( i2\varphi\right) & exp\left( i\varphi\right) \\ exp\left( i\varphi\right) & 2\Delta x \end{matrix} \right)\left( \begin{matrix} 1 \\ 0 \end{matrix} \right)=\left( \begin{matrix} -2\Delta xexp\left( i2\varphi\right) \\ exp\left( i\varphi\right) \end{matrix} \right)\#\left( s9 \right) \end{aligned}$$

$$\begin{aligned} E_{out}^{y}=TE_{y}=\left( \begin{matrix} -2\Delta xexp\left( i2\varphi\right) & exp\left( i\varphi\right) \\ exp\left( i\varphi\right) & 2\Delta x \end{matrix} \right)\left( \begin{matrix} 0 \\ 1 \end{matrix} \right)=\left( \begin{matrix} exp\left( i\varphi\right) \\ 2\Delta x \end{matrix} \right)\#\left( s10 \right) \end{aligned}$$

which can be used to determine the polarization conversion ratio (PCR) as

$$\begin{aligned} PCR=\frac{P_{c}}{P_{uc}+P_{c}}=\frac{\left| E_{c} \right|^{2}}{\left| E_{c} \right|^{2}+\left| E_{uc} \right|^{2}}=\frac{1}{1+\left( 2\Delta x \right)^{2}}=99.99\%\#\left( s11 \right) \end{aligned}$$

where $\Delta x=\pm0.0043(\pm0.25^{\circ})$.

Though the PCR of device can be up to $99.99\%$ in principle, it will further decrease if the angle of alignment error is larger in the practical process, e.g. $PCR=99.88\%$ for $\Delta x= \pm0.0175(\pm1^{\circ})$, $PCR=99.52\%$ for $\Delta x= \pm0.0349(\pm2^{\circ})$, and $PCR=97.04\%$ for $\Delta x= \pm0.0873(\pm5^{\circ})$ as shown in Fig. S5.

The accuracy of alignment orientation is crucial for high PCR performance. Therefore, in order to achieve better PCR performance, the angle error of alignment should be minimized. Photoalignment^1^ has excellent alignment accuracy, and is also compatible with the devices in this article, so this can be used to further improve the performance of PI-LCoS devices in the future.


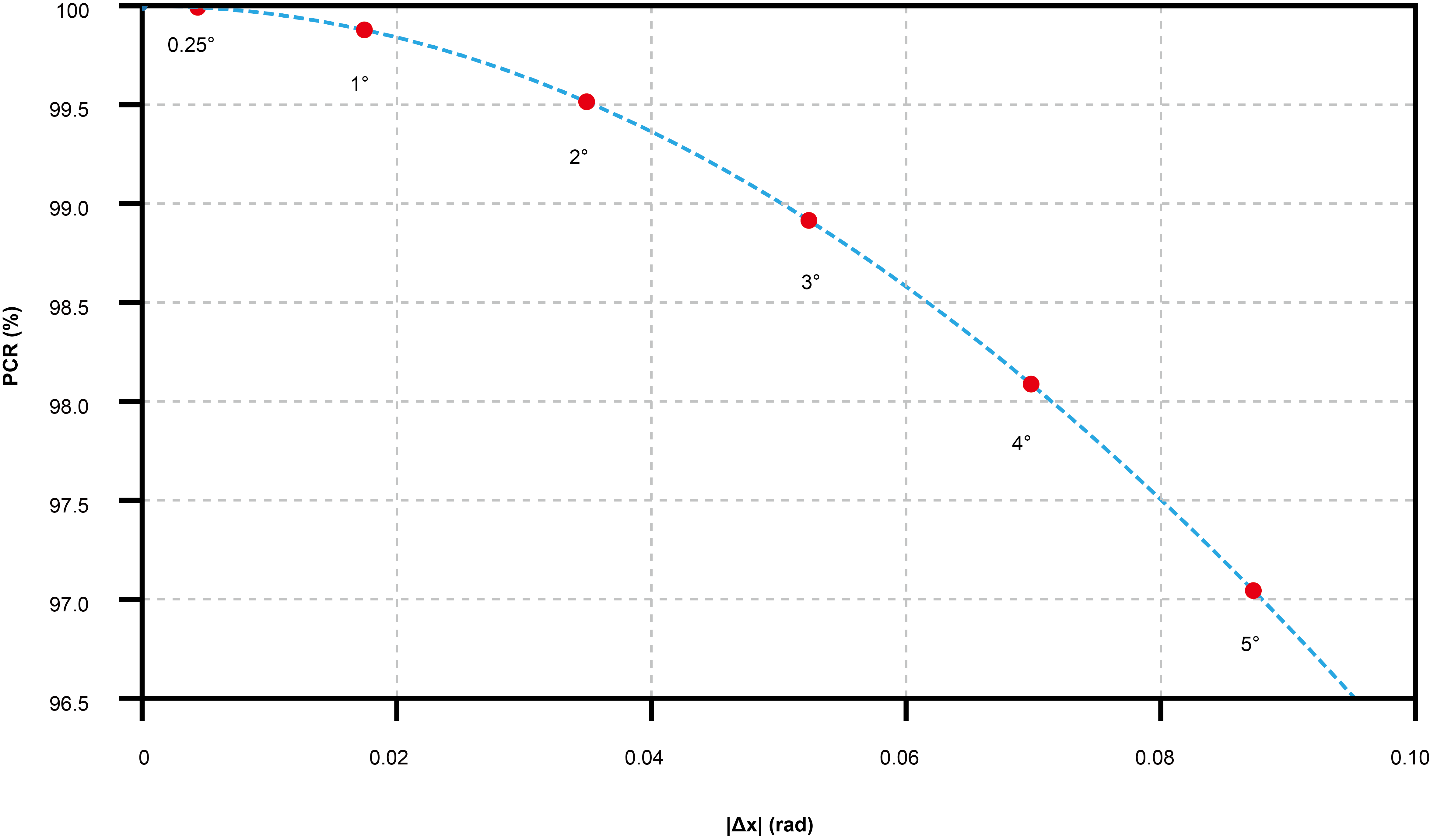


**Fig. S5. Variation in the PCR for device as a function of** $\left| \boldsymbol{\Delta x} \right|$**.** $\left| \Delta x \right|$ is the absolute variation of angle $x$ when $x=\frac{\pi}{4}$.

**Fabrication of metasurface-embedded LCoS devices.** A single-pixel metasurface-embedded LCoS device is firstly fabricated, as shown in Fig. S6, to verify the feasibility of the design scheme, and then a 4K PI-LCoS based on a commercial LCoS backplane is fabricated as shown in Fig. 3 of the main text. The detailed fabrication process as shown in Fig. S7, S8 are described in the “Method” of the main text. Finally, a cross-section SEM image of the single-pixel device as shown in Fig. S9 has been taken to show that the cladding layer fills the entire gap of the nanoantenna arrays properly.


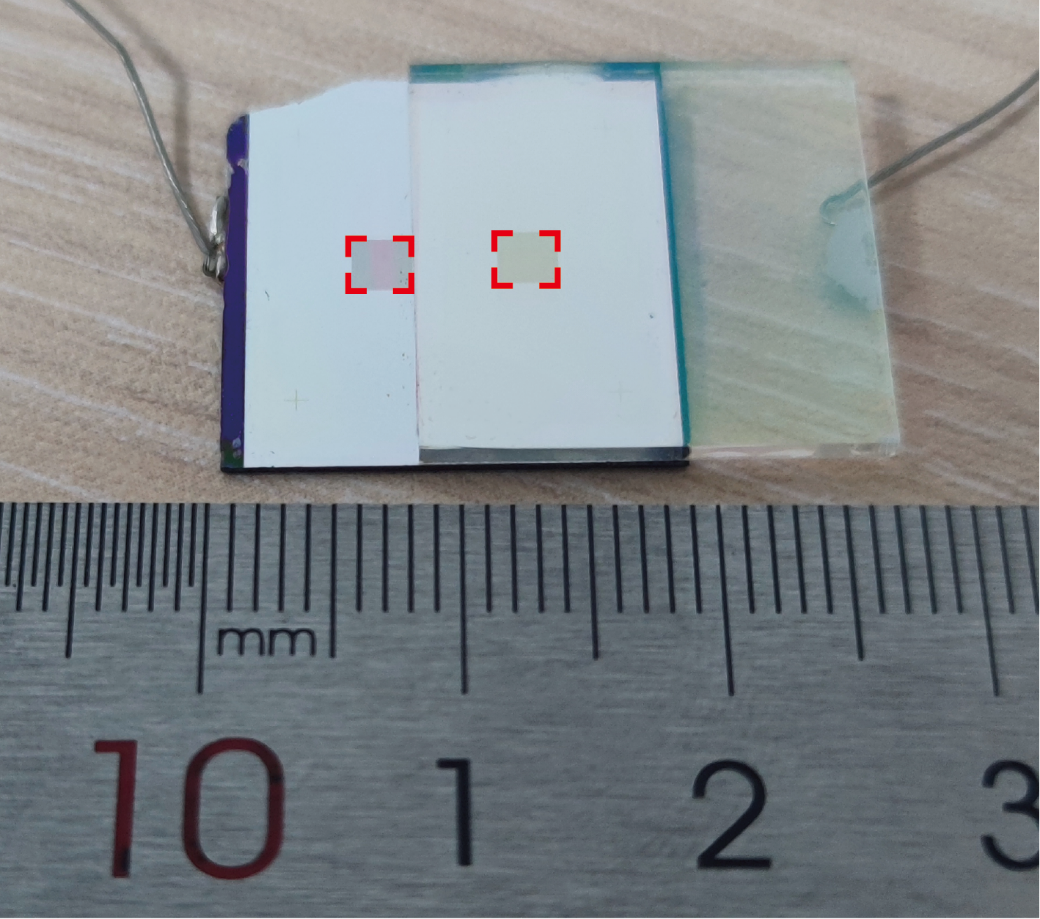


**Fig. S6.** **Image of the fabricated single-pixel metasurface-embedded LCoS device.** Two regions with metasurfaces are indicated by red dashed boxes in size of 2.5 mm × 2.5 mm, one of which has LC packaging while the other has no LC packaging for comparison.


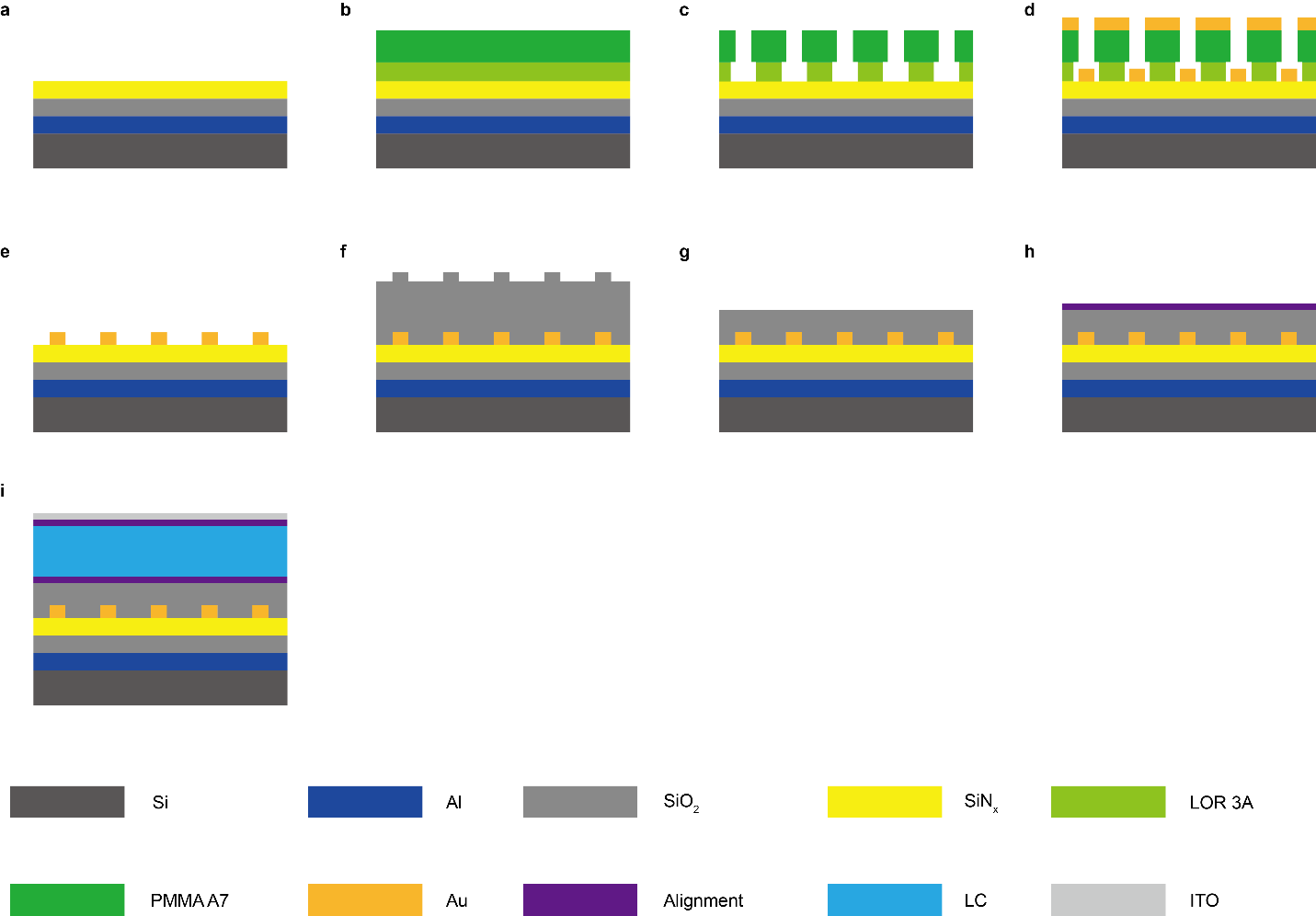


**Fig. S7. Process flow for the fabrication of the metasurface-embedded LCoS device. a** Pristine 4K backplane or the dummy single-pixel substrate formed by depositing Al, SiO_2_, and SiN_x_ films on Si wafer sequentially. **b** Spin coating LOR A3 (200 nm) and PMMA A7 (560 nm) resists in sequence. **c** Pattern on the resist. **d** Deposit Au film. **e** Lift off. **f** Deposit SiO_2_ cladding layer. **g** Etch cladding layer. **h** Spin coating LC alignment film. **i** LC packaging.


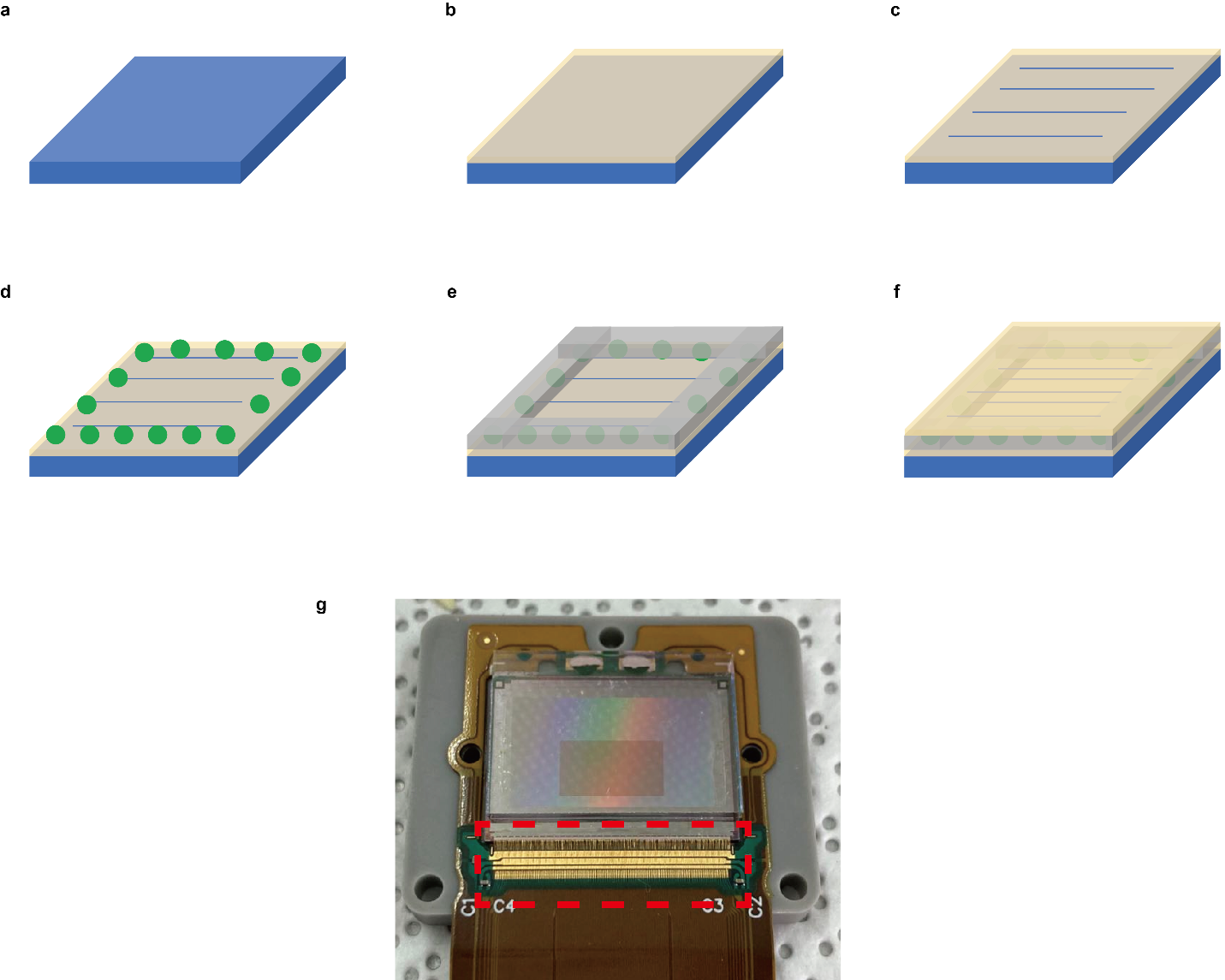


**Fig. S8. LC Packaging.** **a** Clean the device. **b** Spin coating LC alignment layer (Polyimide). **c** Rubbing for alignment. **d** Spray the LC spacer. **e** Coating frame and curing bond. **f** Inject LC and seal. **g** Wire bonding.


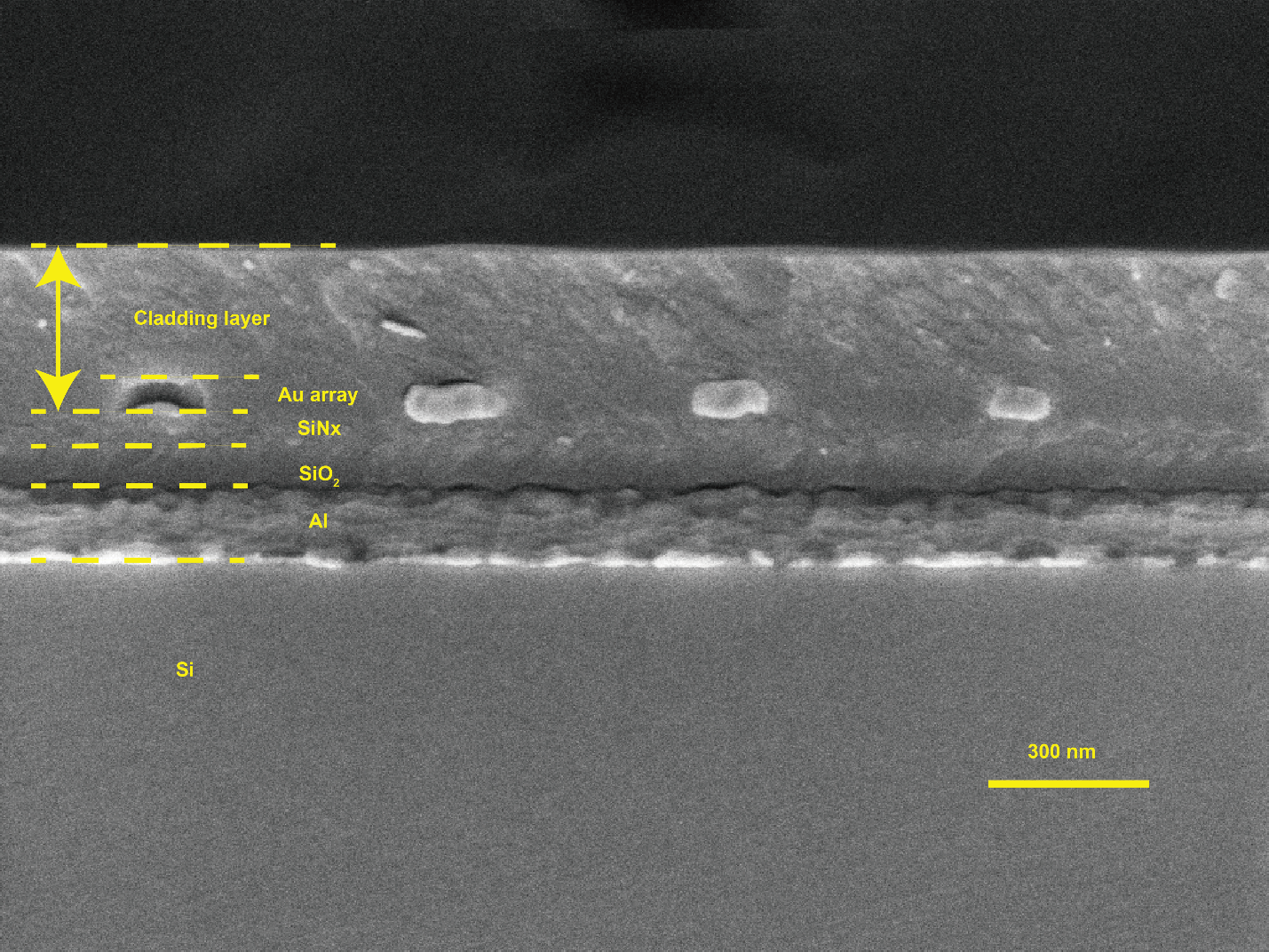


**Fig. S9. SEM image for the cross section of a single-pixel device.**

**Characterization of metasurface-embedded LCoS devices.** Optical setup as shown in Fig. S10 is employed to measure the PCR and electro-optic (EO) response performance of the fabricated metasurface-embeded LCoS devices. The PCR performance of the single-pixel LCoS device is measured before and after LC packaging for comparison as shown in Fig. S11. Noted that both PCR curves are over 93%, which confirms the optimized nanoantenna arrays can maintain high PCR performance even for large change of the surrounding refractive index from 1.0 to 1.8 corresponding to before and after LC packaging, respectively.

The electro-optic phase responses of the original LCoS (the area without metasurface) and the PI-LCoS (the area with metasurface) based on the same single-pixel device are both characterized based on an interference method at the wavelength of 1550 nm. The result (Fig. S12) shows that PI-LCoS has almost the same phase responses for both 0^o^ and 90^o^ linear-polarized light, which verifies the targeted polarization-independent phase modulation. As expected, the amount of phase modulation for the PI-LCoS almost coincides with half of that for the original LCoS, which indicates the embedded ultrathin metasurface structure causes negligible deterioration in the driving voltage.

Based on the measured EO curves, the equivalent refractive index of LC layer can be approximately deduced by $n_{eff}=n_{0}+\left( \Delta\varphi\cdot\lambda\right)/\left( 2\pi\cdot d \right)$, where $n_{0}=1.50$ is the ordinary refractive index of LC, $\lambda=1550 nm$ is the work wavelength, $d=8.5 \mu m$ is the thickness of LC layer, and $\Delta\varphi$ is the difference of the phase modulation amounts corresponding to the target voltage and the reference voltage (3.5 V), respectively~~.~~ The calculated equivalent refractive indices of LC layer are as follows:$n_{eff} \sim1.79 @$0.7 V, $n_{eff} \sim1.70 @$1.0 V, $n_{eff} \sim1.60 @$1.5 V, $n_{eff} \sim1.52@$3.0 V as shown in Fig. 2c of the main text.


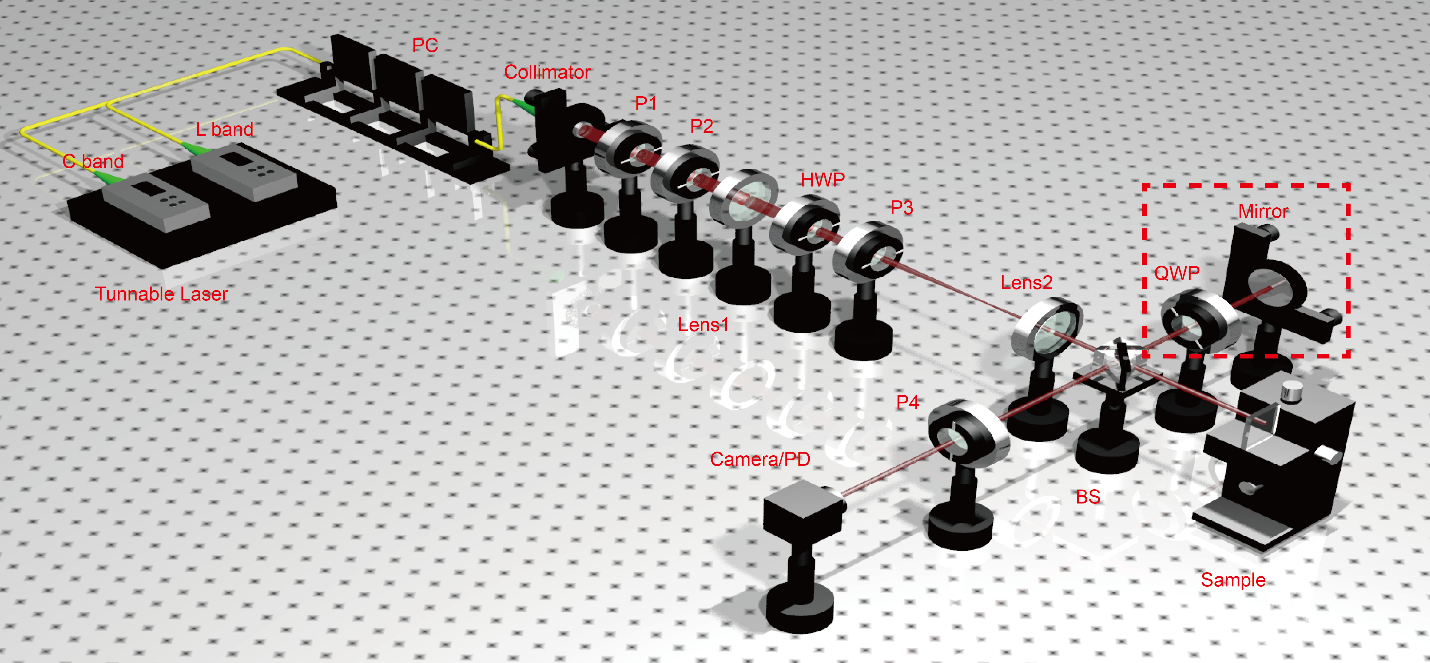


**Fig. S10. Schematic of the optical setup for measuring the performance of the metasurface-embedded LCoS device.** P1-P5: linear polarizers. PC: polarization controller. BS: beam splitter. QWP: quarter-wave plate. HWP: half-wave plate. PD: photodetector. Noted that when adding the QWP and mirrors (the components indicated in the red dashed box), this test system is equivalent to a Michelson interferometer and can be used to measure the phase modulation amount of the metasurface-embedded LCoS device; while removing the components (in the red dashed box), this test system can be used to measure the PCR performance of metasurface-embedded LCoS device.


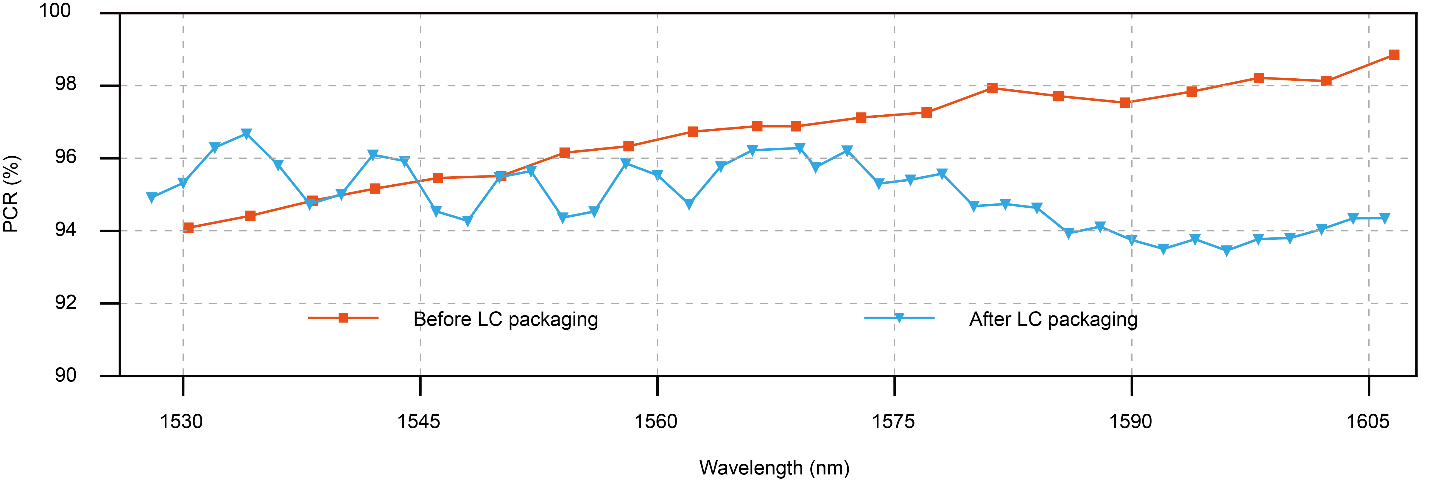


**Fig. S11. PCR performance of the single-pixel metasurface-embedded LCoS device before and after LC packaging.** The measured area corresponds to the metasurface region on the right in Fig. S6.


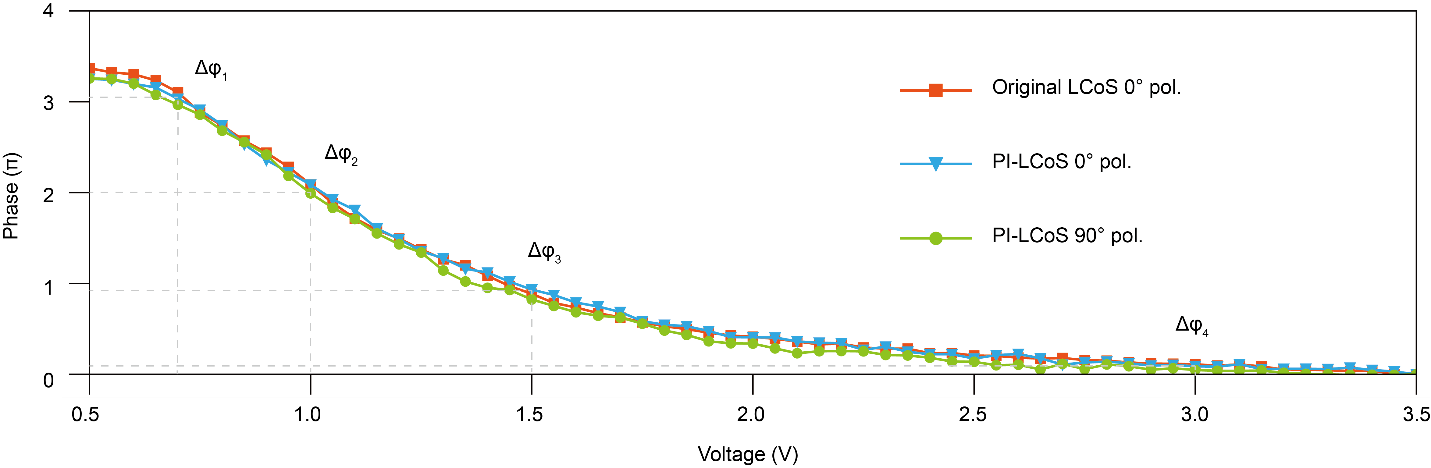


**Fig. S12. Electro-optic phase response of the single-pixel metasurface-embedded LCoS device.** Phase response of the original LCoS (the area without metasurface in the single-pixel device) with 0° (LC alignment direction) linear-polarized incident light and PI-LCoS (the area with metasurface in the single-pixel device) with both 0° and 90° linear-polarized incident light are measured. The amount of phase shift in the original LCoS is halved for better comparison with that of the PI-LCoS.

**Diffraction efficiency of the binary grating.** The binary grating performance is measured and shown in Fig. S13. It is shown that the diffraction efficiency of the binary grating with grating period larger than 6 is very close to the theoretical value of around -4.0dB (~40.5%). As the period further decreases to 4 and even 2, the diffraction efficiency starts to drop to -4.5dB and -6.2dB respectively because of the increasing fringing field effect. However, it should be noted that in consideration of diffraction efficiency, binary grating with the smallest period of 2 will not be applied in real applications as the theoretical diffraction efficiency of 40.5% is too large to be accepted. Typically, blazed grating with period of more than 6 pixels or even 8 pixels will be employed in beam-steering applications such as the commercial WSS modules. Therefore, this work mainly demonstrated the beam steering capability of this device up to 4 degree with blazed grating (grating period of ~6 pixels).


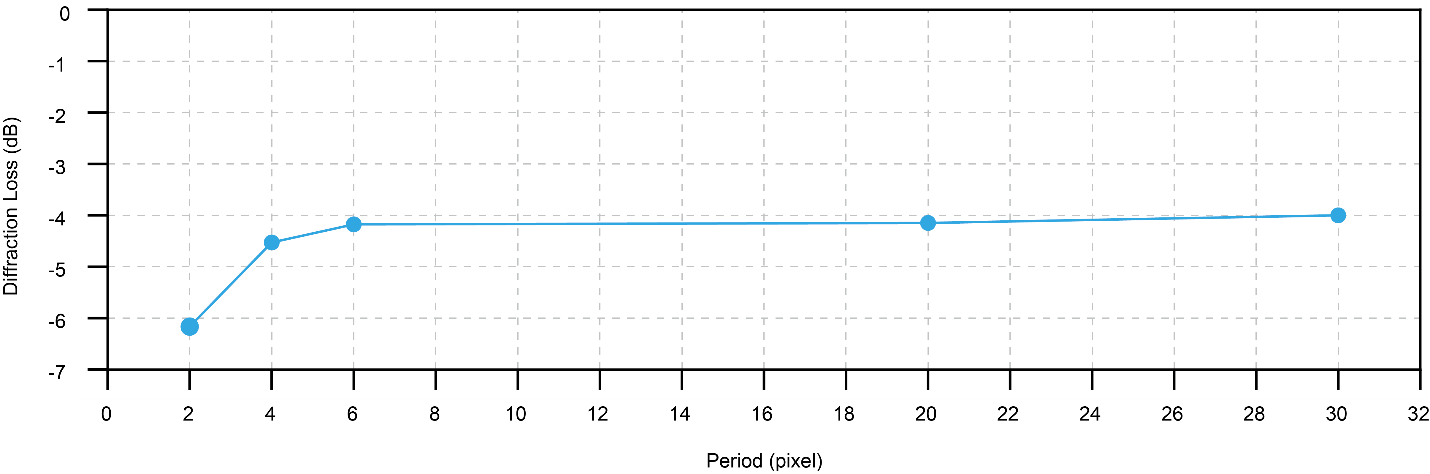


**Fig. S13. Diffraction efficiency of the binary grating with different grating periods.**

**The uniformity of 4K PI-LCoS device.** We use polarized interference microscope to measure the uniformity of the thickness of the LC layer as shown in Fig. S14. It is shown that there is only one newton ring within the large field of view when the PI-LCoS displays a hologram with a uniform graylevel between 0 (minimum) and 255 (maximum), which directly indicates good uniformity of the device. We further characterize the uniformity of the performance by measuring two areas that close to each side of the device. As shown in Fig. S15, both areas of the 4K PI-LCoS maintain high PCR (＞95%) in C+L band, which indicates that the fabricated device also has good uniformity in performance.


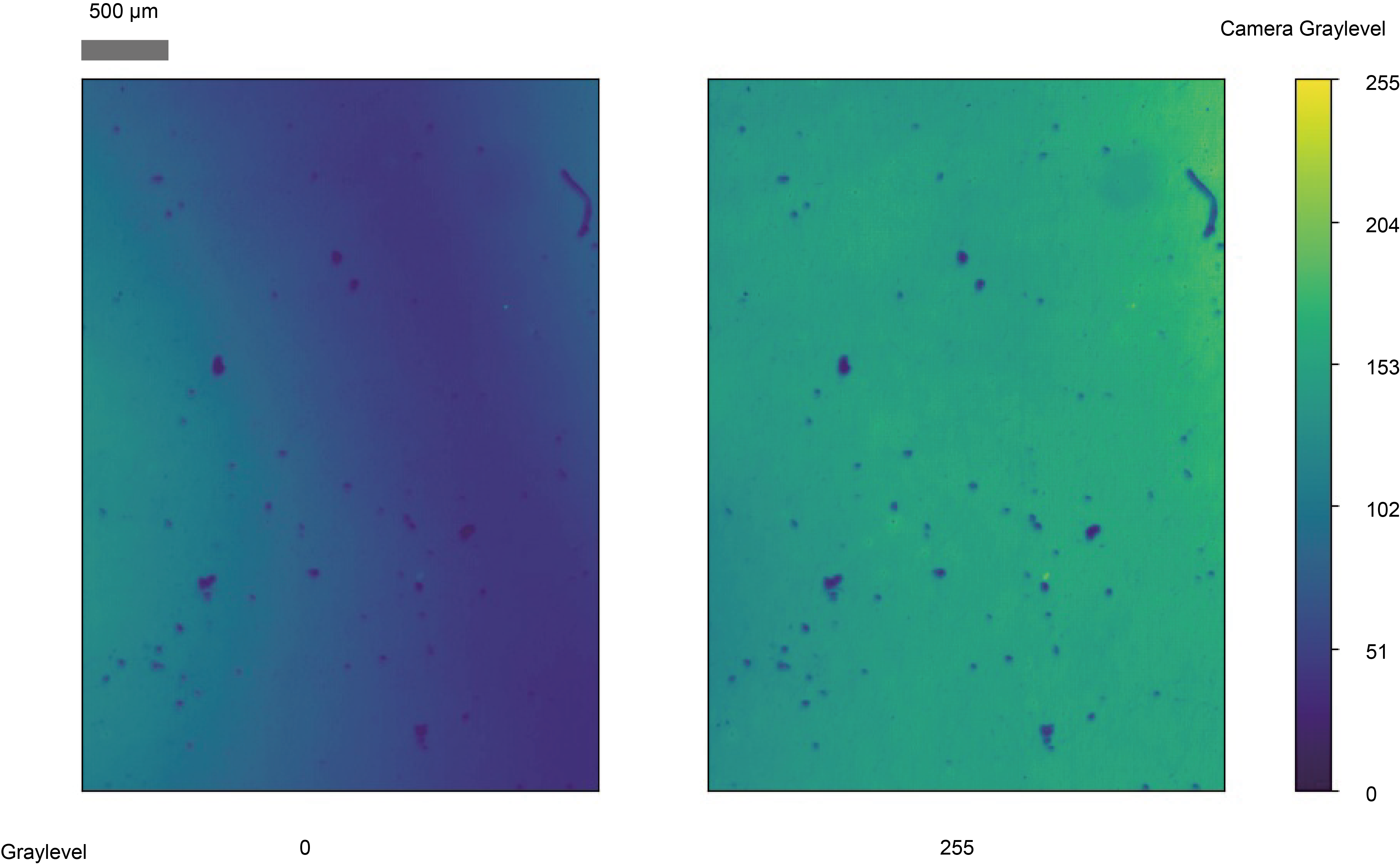


**Fig. S14. The uniformity of polarized interference fringes performance for the 4K PI-LCoS device.**


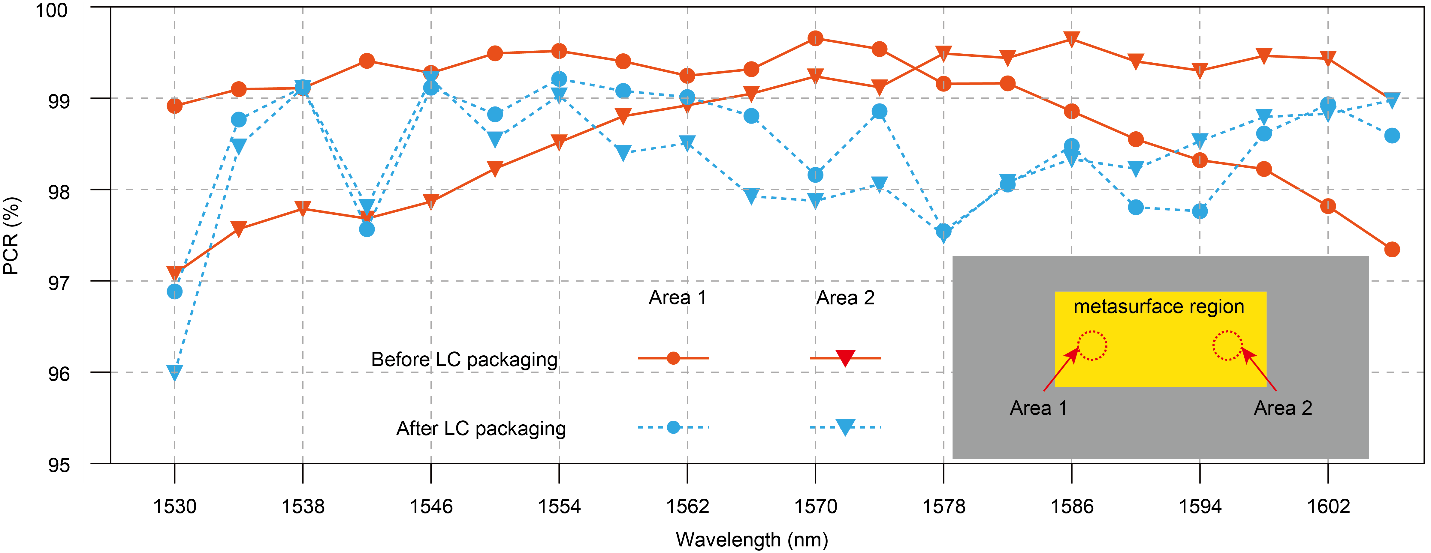


**Fig.S15. The uniformity of PCR performance for the 4K PI-LCoS device.** Area 1/2: two different metasurface area.

**Demonstration of an assembled 1×9 PI-WSS.** The 4K PI-LCoS demonstrated in this work can greatly simplify WSS modules as shown in Fig. S16, where conventional polarization-diversity optics with two separate paths for the two polarizations are no longer needed in the PI-WSS. Instead, both orthogonal polarizations follow a single optical path, significantly eliminating the off-axis effect such that only two cylindrical lenses are required, without additional lenses or wedges for aberration compensation. The simplified and compact design thus greatly reduces the WSS module assembly time by almost 90% from previous 4~6 hours to less than half an hour. Moreover, the assembled 1×9 WSS is characterized with high performance, which is presented in the main text.


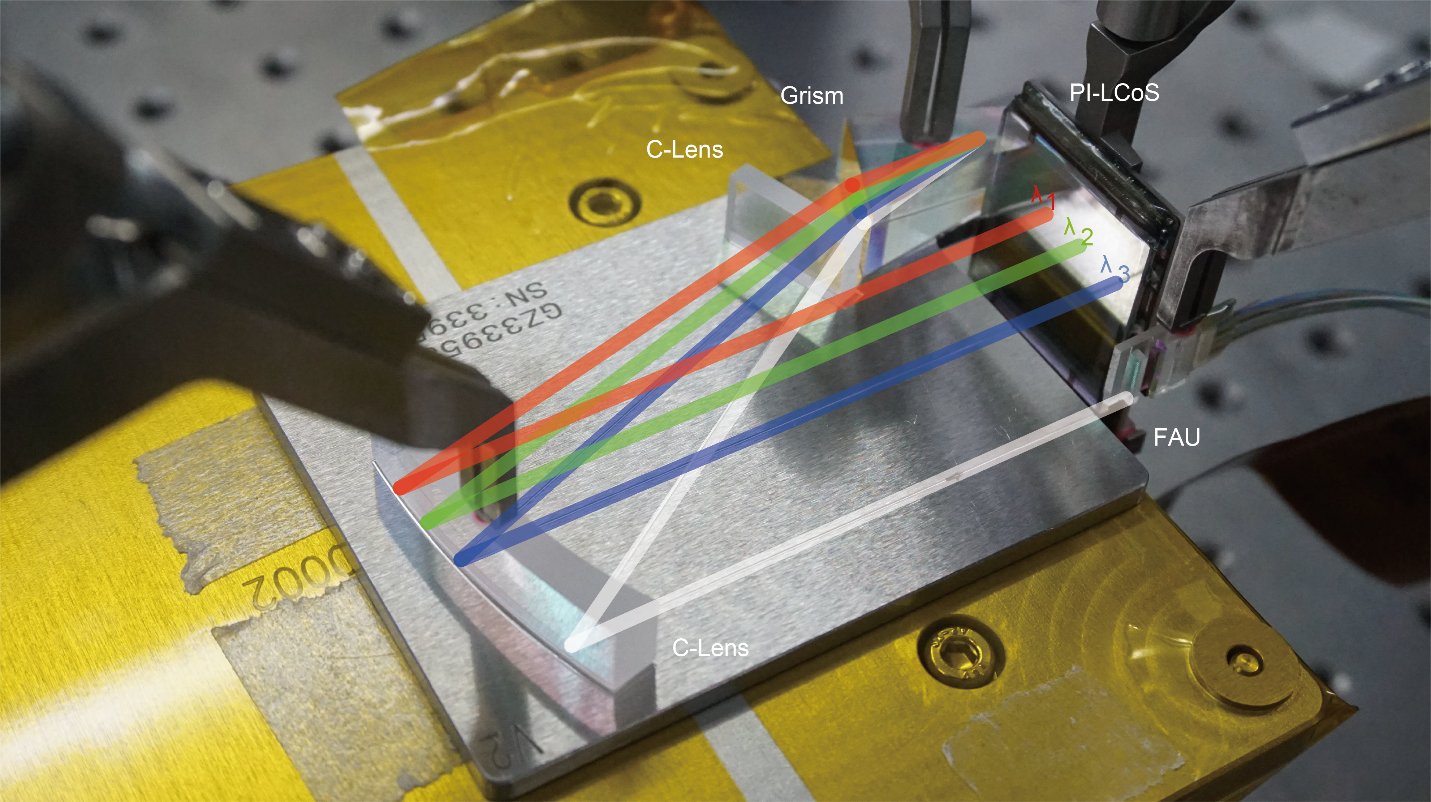


**Fig. S16. Assembled 1×9 PI-WSS based on the 4K PI-LCoS device.** FAU: fiber array unit, C-lens: cylindrical lens, Grism: grating+prism.

**Reference:**

1. Chigrinov, V. G., Kozenkov, V. M. & Kwok, H. S. Photoalignment of Liquid Crystalline Materials: Physics and Applications. (Hoboken: John Wiley & Sons, 2008).
